# How Hydration Weakens Collagen: A Mesoscale Energy Decomposition of Type I and Type II Fibrils

**DOI:** 10.34133/csbj.0050

**Published:** 2026-04-21

**Authors:** Afif Gouissem, Malek Adouni

**Affiliations:** ^1^ Mechanical Engineering Department, Australian University, West Mishref, Kuwait.; ^2^ Biomedical and Instrumentation Engineering, Abdullah Al Salem University, Khalidiya, Kuwait.

## Abstract

This study presents a hydration-dependent mesoscale model to quantify intermolecular cohesive energy in Type II collagen fibrils, the primary structural component of articular cartilage. In contrast to the tightly packed, low-hydrated Type I fibrils, Type II molecules form a loosely organized network in a highly hydrated matrix. Building on Buehler’s coarse-grained framework, we introduced hydration-sensitive corrections and quantified the major contributors to fibrillar cohesion: hydrogen bonding, van der Waals forces, water-mediated bridges, hydrophobic interactions, and coulombic attractions. Quantitatively, the total cohesive energy of Type II collagen was estimated at 2,208 kcal/mol per tropocollagen molecule, substantially lower than 3,046 kcal/mol in Type I collagen. Among the contributing factors, hydrophobic interactions dominated in Type II, accounting for ~52% of the total cohesive energy, followed by coulombic and hydrogen-bonding contributions, whereas van der Waals and water-mediated interactions were substantially weakened by hydration-induced spacing and dielectric screening. To our knowledge, these findings provide the first quantitative energy decomposition of Type II fibrils. They offer insights into the molecular determinants of cartilage flexibility, age-related stiffening, and matrix degradation in osteoarthritis and may help guide the development of biomimetic cartilage constructs.

## Highlights

• A hydration-dependent model quantifies collagen fibrils’ cohesion.
• The first quantitative energy decomposition of Type I and Type II collagen fibrils is presented.
• Type II collagen fibrils’ cohesion is 27% lower than that of Type I.
• Hydrophobic interactions dominate cohesion in Type II collagen fibrils
• The results explain the mechanical softening of cartilage.

## Graphical Abstract

**Figure:**
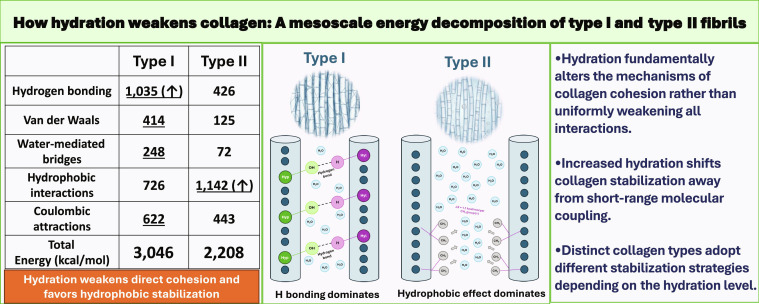

## Introduction

Collagen is the most abundant protein in the human extracellular matrix, comprising over 30% of the total protein content. Among the 28 known isoforms, Type I and Type II collagens are the most structurally and biomechanically important and have been the focus of most collagen research [1–3]. Type I fibrils are tightly packed, providing tensile strength in connective tissues such as tendons and bones [4], whereas Type II fibrils assemble into a loosely packed, highly hydrated network in avascular tissues such as cartilage [5–8].

Although the molecular structure of Type II collagen is based on the same repeating triplets (Gly-X-Y) arranged in the triple-helical conformation observed in Type I, its biochemical environment differs notably. In fact, Type II collagen typically exhibits higher levels of glycosylation, reduced enzymatic cross-linking, and a substantially higher water uptake [9,10]. All these features lead to looser fibril packing and weakened intermolecular interactions [11].

Mechanical data on Type II collagen fibrils remain scarce compared to those on Type I. While experimental studies have reported lower stiffness in cartilage fibrils, these measurements often reflect tissue-level heterogeneity rather than molecular-scale cohesion [12,13]. Furthermore, most molecular-scale insights into Type II mechanics have been inferred indirectly from tissue-level studies [14,15] such as cartilage indentation and swelling studies rather than direct fibril testing. Since direct molecular-scale data are lacking, most modeling studies have focused on Type I collagen. This preference reflects its structural regularity and the availability of experimental data. Atomistic simulations by Buehler [16] revealed key behaviors such as molecular sliding and strain stiffening, which led to coarse-grained models that capture hierarchical stress transfer mechanisms [17,18]. However, these models assume dense fibril packing and strong lateral interactions [19,20], conditions that are incompatible with the hydrated structure of Type II fibrils. Therefore, Type I models cannot capture the mechanical and energetic behavior of Type II collagen.

Recent multiscale efforts have begun analytically characterizing the stress–strain behavior in cross-linked collagen fibrils and benchmarking these against molecular dynamics (MD) simulations [21]. However, despite this progress in Type I, no model has quantitatively decomposed the intermolecular energy landscape of hydrated Type II fibrils into its fundamental components. Consequently, the relative contributions of hydrogen bonding, water-mediated bridges, van der Waals (VdW) forces, electrostatic interactions, and hydrophobic forces remain unquantified. Without mechanistic resolution, our understanding of how water content governs molecular cohesion in Type II collagen is incomplete and the predictive power of current cartilage models remains limited.

Moreover, studies have shown that changes in mineral content [22], collagen cross-linking [23], and advanced glycation end product (AGE) accumulation [24] considerably alter the mechanical behavior of collagen-rich tissues such as ligaments and tendons. This lack of a quantitative mesoscopic model for Type II collagen represents a critical gap in the simulation of cartilage mechanics. Notably, these water-mediated mechanisms are implicated in degenerative conditions such as osteoarthritis, where matrix dehydration and cross-link accumulation compromise mechanical integrity [6]. A quantitative framework that links molecular interactions to macroscopic cartilage mechanics is therefore essential for improving predictive models and guiding tissue engineering strategies.

To our knowledge, this is the first mesoscopic model that quantitatively decomposes the cohesive energy of hydrated Type II collagen into physically distinct components. Building on Buehler’s original bead–spring framework, the resulting model provides a biologically informed, computationally efficient platform for studying collagen fibril cohesion under relevant hydration conditions. It also establishes a foundation for the predictive modeling of cartilage mechanics, aging, and degeneration, as well as the rational design of biomimetic scaffolds.

## Methodology

To quantify the total cohesive energy between molecules in Type I and Type II collagen, we investigated the molecular cohesion mechanisms involved in maintaining the fibril packed in its radial direction. Five principal energy contributions were analyzed and modeled: namely, direct hydrogen bonding, VdW interactions, coulombic interactions, water-mediated hydrogen bridges, and hydrophobic energy associated with water organization.

These categories represent the major intermolecular forces known to contribute to the lateral stabilization of collagen fibrils. Other forces, such as π–π stacking, were excluded because they are absent from the molecular structure of collagen or contribute negligibly to fibrillar cohesion. This decomposition is supported by experimental and computational studies [25–27], as well as recent works analyzing multiple interaction mechanisms in collagen fibrils [28,29] and identifying these 5 interaction types as dominant contributors to molecular-level cohesion and resistance to sliding between collagen molecules. Each interaction class was parametrized using molecular structural data, statistical occurrences, electrostatic parameters, and distance-scaling factors to account for geometric differences as detailed in subsequent sections. Longitudinal interactions were not analyzed or quantified in this study.

The influence of amino acid composition is implicitly incorporated in this framework through the statistical composition of the collagen sequence. Residue counts obtained from the UniProt sequences of Type I and Type II collagen [30] were used to determine the frequencies of the functional groups participating in the different interaction mechanisms considered in this study. In this way, the chemical diversity of residues contributes to the interaction statistics without modeling site-specific residue contacts. Because each tropocollagen (TC) molecule contains several thousand residues and forms a large number of intermolecular contacts, the present mesoscale model focuses on the global average cohesive energy rather than residue-level interactions, consistent with coarse-grained modeling approaches commonly used for collagen fibrils [18].

Different classes of amino acid residues contribute to distinct interaction mechanisms within the fibril. Hydrophobic residues promote cohesion through CH_2_/CH_3_-mediated interactions associated with water exclusion, while polar residues act as hydrogen-bond donors and acceptors, and charged residues contribute to electrostatic stabilization. The effectiveness of these interactions is strongly modulated by hydration, with low hydration in Type I collagen favoring direct hydrogen bonding and electrostatic interactions and high hydration in Type II collagen leading to reduced direct interactions and increased contribution from water-mediated and hydrophobic mechanisms.

This decomposition allows each force category to be quantified separately based on established physical principles. Hydration levels, treated as parameters, allowed comparison of the cohesion dominant mechanisms in Type I and Type II fibrils.

### Overview of cohesive energy calculations

For each interaction mechanism, the cohesive energy was computed using the same 2-step general procedure. First, the number of intermolecular interactions per TC molecule was estimated from the molecular structure of the fibril, the amino acid composition of the collagen molecule, and structural information reported in previous studies. Second, a representative energy value was assigned to each interaction type based on experimental measurements or molecular simulations reported in the literature. The total energy contribution of each mechanism was then obtained by multiplying the number of interactions by the corresponding interaction energy.

#### Hydrogen bonding

For Type I collagen, the number of intermolecular hydrogen bonds per TC molecule was obtained from MD simulations reported by Streeter and de Leeuw, which identified approximately 392 stable collagen–collagen hydrogen bonds between neighboring molecules. In the simulations of Streeter and de Leeuw, hydrogen bonds were identified using standard geometric criteria (donor–acceptor distance < 3.2 Å and donor–H–acceptor angle > 150°), consistent with MD conventions.

For Type II collagen, the number of bonds was estimated using a Hill-type binding relation accounting for the higher hydration level and the higher competition between water molecules and collagen binding sites. The energy per hydrogen bond was assigned using MD results reported by Sheu et al., which provide hydrogen-bond strengths for peptide systems under different hydration conditions. For Type II collagen, the bond energy was taken directly from the value reported for highly hydrated peptide systems. For Type I collagen, the bond energy was estimated by interpolating between the dehydrated and hydrated limits reported in these simulations.

#### Water-mediated hydrogen bridges

The number of water-mediated bridges per TC molecule was estimated by assuming one single-water bridge per Gly-X-Y triplet of the collagen sequence, based on density functional theory (DFT) studies of collagen-like peptides and structural analyses of hydrated fibrils. The interaction energy of each bridge was assigned from crystallographic analyses of protein interfaces reporting an average energy of 0.46 kcal/mol for single-water bridges. For Type II collagen, the bridge energy was adjusted by transforming single-water bridges into double-water bridges to account for the increased intermolecular spacing caused by hydration and applying an inverse-power distance-dependent decay $1/r^{3}$ to the interaction energy.

#### VdW interactions

VdW cohesion was estimated by computing contacts between surface-accessible side-chain atoms and assigning interaction energies using a Lennard-Jones (LJ) potential. The atomic composition of each TC molecule was obtained from the UniProt sequences of Type I and Type II collagen. The total number of VdW contacts per molecule was determined by averaging the stoichiometric frequencies of surface-exposed atom types (C, H, O, N, and S) and calculating their probable pairwise interactions across the hydration-dependent intermolecular gap. The interaction energy for each atom pair was computed using LJ parameters from the CHARMM36 force field. For Type II collagen, interatomic distances were increased using a hydration-induced swelling factor of 1.4, calculated from the relative increase in intermolecular spacing reported for hydrated collagen fibrils, and applied to side-chain atoms.

#### Hydrophobic interactions

Hydrophobic cohesion was estimated using the amino acid composition of Type I and Type II collagen sequences, by counting nonpolar CH_2_ and CH_3_ side-chain groups capable of displacing structured water upon intermolecular contact. The interaction energy of each group was assigned using Pace et al.’s thermodynamic formulation for hydrophobic burial, in which each CH_2_/CH_3_ group contributes an entropic stabilization in hydrated environments. For Type I and Type II collagen, the energy per group was scaled according to the respective hydration level of the fibril.

#### Coulombic interactions

Coulombic cohesion was estimated by considering short-range electrostatic interactions between oppositely charged side chains. Only the charged residues were included as potential interaction sites. The number of interactions was calculated statistically from the frequency of charged residues and the number of geometrically accessible neighboring residues across adjacent TC molecules. The interaction energy of each pair was assigned using representative salt-bridge energies reported by Nayek et al. from Poisson–Boltzmann electrostatic analyses of protein structures, corresponding to typical contact distances of 3 Å. For Type II collagen, the interaction energy was scaled according to the ratio of the effective dielectric constants of Type I and Type II fibrils.

### Hydrogen-bonding energy

Hydrogen bonding plays a key role in maintaining the lateral cohesion between TC molecules [19]. These bonds can either form between backbone carbonyl (C=O) and amide (H–N) groups or between hydroxyl-bearing (–OH) side chains from adjacent molecules [1]. The frequency and strength of these bonds are highly dependent on the tissue hydration level. For example, in low-hydration tissues such as tendons, hydrogen bonds are highly stabilized by the low-dielectric medium [4,5]. In contrast, in highly hydrated tissues, such as cartilage, water molecules shield direct hydrogen bonding and promote water-mediated bridges [19]. In this section, we aim to quantify the number of stable direct hydrogen bonds per TC molecule, as well as the energy contribution for each bond in Type I and Type II collagen.

**Fig. 1.**
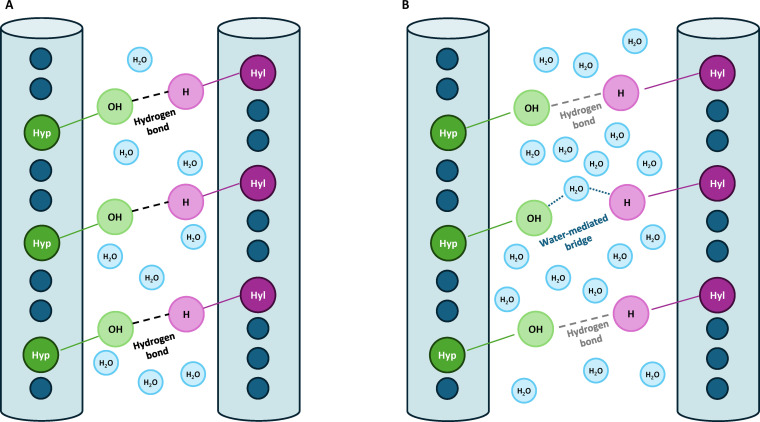
Comparison of intermolecular hydrogen bonding in collagen fibrils. The H and OH groups extending from each cylinder represent the ends of side chains that project outward from the triple helix to form hydrogen bonds. (A) Type I collagen: closer molecular spacing allows multiple direct hydrogen bonds. (B) Type II collagen: higher hydration increases spacing and introduces water-mediated bridges, reducing the number of direct bonds.

As illustrated in Fig. 1, several structural features influence the formation and stability of hydrogen bonds. In particular, the geometric differences between Type I and Type II collagens lead to distinct hydrogen-bonding behaviors [31]. While hydration is the key factor primarily due to the water molecules shielding potential bonding sites, other relevant variables include differences in packing density, which affect intermolecular distances, and the degree of glycosylation, where additional glycosylated residues can attract water molecules, altering bonding patterns. Type II collagen has a higher hydration ratio, looser lateral packing, and increased glycosylation, all of which can restrain hydrogen-bond stability and accessibility. Because hydration has the most direct and quantifiable effect on stable hydrogen-bond formation, we treat it as the dominant parameter in our analysis.

In Type I collagen, hydration is well-defined: tissue contains 1.6 g of H_2_O/g of dry collagen [32], corresponding to 61.5% water by weight. This hydration consists of approximately 0.8 g of H_2_O/g of dry collagen tightly bound primary water and 0.8 g of H_2_O/g of dry collagen loosely bound secondary water [32–34]. Type II collagen contains considerably more water, ranging from 67% to 74% by weight depending on the depth within the cartilage tissue [35]. In this study, we use an average hydration value of 70.5%, corresponding to 2.39 g of H_2_O/g of dry collagen (a 49% increase in water volume compared to that for Type I collagen). Assuming that the volume increase is due only to the added water molecules in Type II collagen, we calculated the lateral hexagonal lattice distance to be 19.1 Å (versus 16.52 Å in Type I collagen [19]). The plausibility of this assumption is supported by Grynpas et al. [36], which proves that upon complete dehydration, both Type I and Type II collagen fibrils collapse to a common spacing of 12 Å, indicating that differences in lateral spacing are indeed driven by water content. Detailed calculation of water content and lattice constants for both collagen types are presented in Appendix A.

While the above analysis relates the increase in lattice spacing primarily to hydration effects within the fibril, it is important to clarify that the present model focuses exclusively on intermolecular interactions within the collagen fibril. Other factors, such as proteoglycans and their associated glycosaminoglycan chains in cartilage, are known to generate osmotic swelling pressures and contribute to the overall volume expansion of the extracellular matrix. However, these macromolecules are primarily located in the extrafibrillar matrix and therefore influence tissue-level swelling rather than the intrinsic molecular packing within collagen fibrils [37–39]. The lattice expansion is therefore restricted to the hydration-induced increase in intermolecular spacing within the fibril itself, which is represented here by a radial expansion between adjacent TC molecules. This assumption reflects the anisotropic nature of fibril swelling, where hydration primarily increases lateral intermolecular spacing, while the axial length of the triple helix remains unchanged [40].

This expansion in lattice spacing reflects not only increased total water content but also a structural rearrangement in how water is distributed within the fibril. Structural studies show that the tightly bound hydration shell on the collagen molecule remains conserved, as does the triple-helix core itself, which maintains a diameter of 10 Å under varying hydration states [41]. The additional water is then loosely bound, forms an additional layer, and increases the extrafibrillar distance. Consequently, the number of stable direct hydrogen bonds between adjacent Type II collagen molecules is reduced compared with that of Type I.

MD simulations by Streeter and de Leeuw [25] reported that each Type I TC molecule forms approximately 5,017 hydrogen bonds in total, only 392 of which bond with neighboring collagen molecules, where the remaining 4,625 bond with surrounding water molecules. In the MD simulations of Streeter and de Leeuw, hydrogen bonds were identified using standard geometric criteria consisting of a donor–acceptor distance cutoff of 3.2 Å and a donor–H–acceptor angle greater than 150°. The reported value of 392 therefore corresponds specifically to all intermolecular collagen–collagen hydrogen bonds between adjacent TC molecules without distinguishing between backbone or side-chain groups.

This number is consistent with chemical expectations, given that a Type I TC molecule consists of 3,233 amino acids based on UniProt sequence data [30], each providing 1 to 2 polar donors or acceptor sites (detailed amino acid counts for both collagen types are presented in Appendix B). Most of these potential binding sites are saturated by water molecules, thereby preventing the formation of direct hydrogen bonds. Only 7.8% of these bonds are protein–protein bonds directly connecting the 2 adjacent molecules, while the vast majority are transient protein–water interactions. We therefore consider 392 stable collagen–collagen bonds per TC molecule for Type I collagen.

For Type II collagen, we estimate the number of direct hydrogen bonds by using Hill’s equation, widely used in understanding binding processes in biological systems [42,43]. This method allows us to describe the competition between water molecules and neighboring collagen side chains in filling the potential binding sites. The percentage of binding sites filled by water molecules is therefore given by

$$\theta_{\mathrm{II}}=\frac{C_{\mathrm{II}}^{n}}{K_{d}^{n}+C_{\mathrm{II}}^{n}}=\frac{2.4^{1}}{2.4^{1}+0.136^{1}}=94.5\%$$ (1)

where $\theta_{\mathrm{II}}$ is the fractional occupancy of sites by water for Type II collagen, $C_{\mathrm{II}}$ is the water concentration (g H_2_O/g dry collagen), n=1 is Hill’s cooperative index indicating no cooperative binding, and $K_{d}=0.136$ is a dissociation constant, fitted using the water concentration and fractional occupancy for Type I collagen. Using the total number of bonds [25] N=5,017, the total number of hydrogen bonds in Type II collagen is obtained:

$$n_{\text{Hydrogen}\_\mathrm{II}}=\left(1-\theta_{\mathrm{II}}\right)*N=269\;\text{bonds}$$ (2)

This reduction is consistent with the greater shielding by water molecules and the increased intermolecular spacing in Type II collagen.

Several studies attempted to measure the hydrogen-bond strength in different biological structures. For example, MD simulations by Sheu et al. [44] reported a hydrogen-bond strength of 1.58 kcal/mol for a β-hairpin peptide solvated in 485 water molecules (corresponding to 6.6 g of H_2_O/g of dry protein). The simulation considered only direct hydrogen bonds and excluded water-mediated bridges, which is consistent with our methodology. Therefore, we adopted this value as an estimate for Type II collagen under physiological hydration. Although this hydration level is substantially higher than that of Type II collagen (2.39 g of H_2_O/g of dry collagen), most of the excess water in the simulation corresponds to bulk solvent. As a result, the marginal weakening effect of additional water diminishes, and the energetic contribution of direct peptide–peptide interactions asymptotically approaches a minimum. Thus, the 1.58 kcal/mol value serves as a conservative, lower-bound estimate for structurally persistent hydrogen bonds in highly hydrated fibrils [45].

In contrast, Sheu et al. [44] measured the protein–protein hydrogen bond in dehydrated systems as 4.79 kcal/mol. This value does not represent the energy in Type I collagen since it contains 1.6 g of H_2_O/g of dry collagen. Using Sheu et al.’s vacuum-phase energy of 4.79 kcal/mol as the dry-state upper bound, a linear extrapolation was applied to estimate the hydrogen-bond energy at this intermediate hydration level. This yielded a value of 2.64 kcal/mol per bond, reflecting reduced water competition and stronger peptide–peptide interactions.

While the hydrogen-bonding estimates are supported by MD and experimental data, certain limitations should be acknowledged. The use of a linear interpolation to estimate hydrogen-bond energies at intermediate hydration levels is a first-order approximation based on the principles of dielectric screening. Physically, this approach assumes that the local dielectric constant of the interfibrillar medium scales with the water density between the collagen chains.

While the fundamental coulombic force follows a 1/*ε* dependence, the transition of the local environment from a low-dielectric protein core to a hydrated interfacial phase involves complex solvent-shielding effects and the formation of ordered water structures. In the absence of high-resolution experimental curves for these confined geometries, this linear energetic interpolation serves as a standard and justifiable baseline that captures the transition from vacuum-like bond strengths to fully solvated states. The validity of this assumption is further supported by the resulting energy of 2.64 kcal/mol, which aligns closely with the weighted averages of direct and water-mediated bond energies reported in independent crystallographic studies of collagenous proteins, despite discrepancies among studies: Jenkins et al. [46] measured a bond contribution of 2.0 kcal/mol in hydrated collagen-mimetic peptides, while Boryskina et al. [47] reported values between 1.4 and 1.8 kcal/mol depending on hydration and packing. Together, these findings support a hydration-dependent framework in which hydrogen-bond energies range from 1.58 to 2.64 kcal/mol for Type II and Type I collagen.

Having obtained the number of stable hydrogen bonds as well as their respective energy contributions, we obtained a total cohesive energy of 1,035 kcal/mol for Type I collagen versus 426 kcal/mol for Type II collagen. These values reflect the hydration-based differences between fibril types and are used as the hydrogen-bonding parameters in the mesoscopic interaction model.

Additional validation for our hydrogen-bond estimate is provided by experimental calorimetric data. Zhang et al. [48] reported a total hydrogen-bond energy of 17.74 J/g for Type I collagen, based on both a mathematical model and differential scanning calorimetry results. Assuming a molecular weight of 300 kDa per TC molecule, we obtained total energy of 1,270 kcal/mol. This experimentally derived value aligns closely with our modeling estimate, supporting its use in our framework.

While the hydrogen-bonding estimates are strongly supported by MD and experimental data, certain limitations should be acknowledged. First, the linear interpolation of hydrogen-bond energies between dehydrated and hydrated systems is a simplified approach that does not fully capture the nonlinear effects of hydration and the dynamics of the local molecular environment. Second, local variations in cross-link density and glycosylation could lead to deviations in the number of direct hydrogen bonds derived from statistical averages. Finally, our analysis focuses solely on structurally stable hydrogen bonds, excluding short-lived interactions that may also contribute to the overall cohesive behavior. Despite these limitations, the close agreement between our model predictions and calorimetric data supports the reliability of the reported energies as estimates of the hydrogen-bonding contribution to the overall cohesive process.

### Water-mediated bridges

**Fig. 2.**
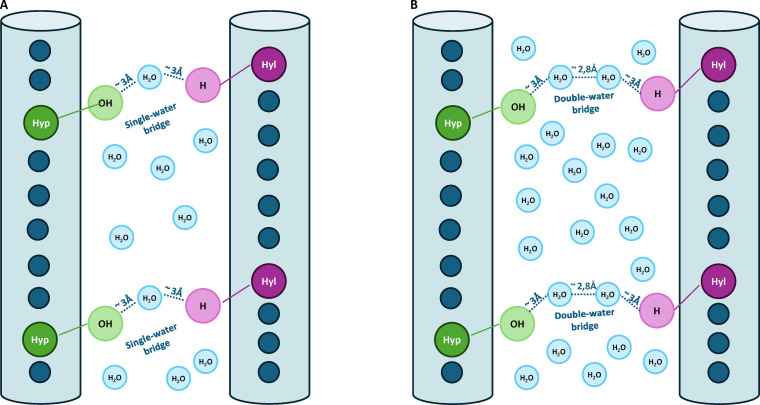
Comparison of intermolecular water-mediated bridges in collagen fibrils. (A) Type I collagen: single-water bridges connecting hydroxyl (Hyp) and hydrogen (Hyl) sites across adjacent molecules, typically spanning a total distance of ~4.92 Å. (B) Type II collagen: increased intermolecular spacing and higher hydration promote the formation of double-water bridges, extending the total interaction length to ~7.72 Å and reducing direct contact between side chains.

The presence of water molecules in collagen fibrils plays a key stabilizing role by mediating interactions between adjacent TC molecules. These water bridges complement the direct peptide–peptide hydrogen bonds discussed in the previous section by connecting hydrogen donors and acceptors on neighboring TC molecules through water molecules, thereby forming water-mediated bridges. Two common types are single-water bridges (H–H_2_O–OH) and double-water bridges (H–H_2_O–H_2_O–OH), which differ in length and interaction strength [49] as illustrated in Fig. 2.

Following the same methodology used in the hydrogen-bond evaluation, a 2-step process was employed. First, the number of water-mediated hydrogen bonds per TC molecule was estimated based on experimental, structural, and statistical analyses. Second, representative interaction energies were drawn from quantum-mechanical calculations and MD simulations.

The reported number of water-mediated bridges in collagen fibrils varies notably across studies, largely due to methodological differences in resolution and hydration modeling. Techniques including DFT [50], MD simulations [20,51], crystallographic interface surveys [52,53], and atomic force microscopy [54] provide complementary yet sometimes divergent perspectives. The variation in reported water-mediated bond counts is largely due to the definition and detection differences across methods. For example, DFT resolves locally stable single-water and occasional double-water bridging geometries at specific sites. In contrast, MD simulations incorporate dynamic hydration shells and transient bonding events that may persist for only picoseconds [55,56], leading to higher total counts.

To establish the number of water bridges, we used a structurally grounded approach by investigating DFT studies specific to collagen-like peptides. Cutini et al. [50] demonstrated that stable single-water bridges form between backbone polar groups in well-defined geometries, consistent with the axial translation of the triple helix of 0.90 nm per Gly-X-Y triplet [41,57] and with interface surveys indicating that single-water bridges predominate over multiwater chains [52,53]. Guided by this evidence, we assumed an average of one water bridge per triplet to represent the overall interaction density throughout the fibril. We acknowledge that real fibrils are not perfectly uniform; they have a D-periodic structure with alternating “gap” and “overlap” regions that hold different amounts of water. However, since our goal is to compare the total bonding strengths of Type I and Type II collagen, using an average count per triplet provides a consistent and practical way to measure the total energy. This approach captures the bulk effect of hydration on the whole structure without needing to map out every local fluctuation in the molecular gaps. Using the amino acid sequences in Appendix B, this corresponds to 1,078 bridges per TC in Type I and 1,216 in Type II.

As validation to this estimate, results were compared against MD data. Streeter and de Leeuw [25] reported a total of 4,625 bonds involving water molecules. This number includes both water–collagen and water–water contacts without explicitly distinguishing water-mediated bridges between adjacent collagen molecules from nonbridging water–water interactions. On the other hand, Ahmed et al.’s work [52] analyzed 179 crystallographic protein interfaces and reported that only 21% of interfacial waters act as bridges, while the rest act as transient molecules that do not participate in stable bridging interactions. Applying this percentage to Streeter and de Leeuw’s dataset yields 971 bridges per TC molecule. This value is in close agreement with the DFT-based estimate (1,078 for Type I), lending confidence that the adopted one-bridge-per-triplet assumption provides a reliable and representative measure of water-mediated bonding in collagen fibrils.

To quantify the cohesive strength of single-water bridges in Type I collagen, we adopted an average value of 0.46 kcal/mol per bridge reported by Ahmed et al. [52] from crystallographic analysis of protein–protein interfaces. Because crystallographic datasets are dominated by single-water bridges [58,59], this value primarily reflects such interactions, making it a reasonable assumption. This estimate is consistent with bond energetics reported in peptide systems ranging from 0.5 to 1.5 kcal/mol [60]. Additional support comes from spectroscopic studies by Park et al. [61], which show that water molecules at protein surfaces exhibit free-energy shifts of approximately 0.4 kcal/mol relative to bulk water, confirming the reduced strength of interfacial hydrogen bonds.

For Type II collagen, the extra hydration plays a role in increasing the interatomic distances and thus decreasing the overall cohesion energy. Since the triple-helix core has already been established to maintain a fixed diameter of ~10 Å under varying hydration states [41,57], 2 modeling strategies can be adopted. In the first, this conserved core geometry implies that the observed increase in fibril diameter must originate entirely from the expansion of the interstitial space between adjacent molecules. Therefore, a uniform swelling factor of 1.40 was applied to all interatomic distances outside the triple helix. Detailed calculations of the swelling factor are provided in Appendix C. As hydrogen-bond strength decreases with increasing distance, this model predicts a decay in interaction energy without altering the bridge types. Although this approach is based on solid geometric assumptions, it fails to explain the actual bonding mechanisms in Type II collagen.

In the second approach, we propose that hydration-driven swelling leads to the structural elongation of the bridges themselves: existing single-water bridges in Type I become double-water bridges in Type II [36,54,62]. This transformation reflects the insertion of an additional water molecule layer between TC molecules, increasing the donor–acceptor distance by one water–water (O_w_–O_w_) spacing. This second assumption also aligns with experimental observations of an increased fibril diameter in Type II collagen, approximately 2.8 Å larger than that in Type I, matching the size of the additional bridging water layer. This model provides a physically plausible and biologically grounded basis to evaluate the effect of hydration on water-dependent cohesion.

Following either approach, the energy estimation is solely based on the interatomic distances between the donor and the acceptor. Therefore, as a reference point, the bond distances for a single-water bridge were calculated: the donor–H–O_w_ and O_w_–H–acceptor leg distances typically span 2.8 to 3.0 Å [26,63]. This is in agreement with the collagenous peptide distances extracted from 1CGD and 1GAC structures [41,57], which represent stabilized triple helices in structured collagen environments. Analysis of these structures revealed an average leg length of approximately 3.02 Å and an average donor–acceptor distance of 4.92 Å.

For double-water bridges (donor–H–O_w1_–O_w2_–H–acceptor), the donor–acceptor distances were calculated by summing the single-water bridge distance (4.92 Å) with the typical O_w1_–O_w2_ spacing of 2.8 Å [26,63], giving a total path length of ~7.72 Å. To account for the reduced interaction strength over distance, we apply a $1/r^{3}$ distance-dependent decay function, as predicted by classical electrostatics for dipole–dipole interactions [64]. This scaling reflects the physical basis for polarization-mediated coupling across solvent-separated regions. Applying this scaling, the interaction energy of a double-water bridge is reduced to a factor of 0.26, or 74% lower than that of a single-water bridge, purely due to geometric separation. Using the reference energy of 0.46 kcal/mol [52], this yielded an effective contribution of 0.12 kcal/mol for a double-water bridge. This estimate is consistent with prior observations that long-range, water-mediated bonds contribute weakly to protein and fibrillar stability [53,61,65].

Despite their conceptual differences, the 2 modeling strategies yield results that differ only by 7 kcal/mol per Type II TC molecule. This variation represents a negligible fraction of the total cohesive energy of the fibril, which reaches several thousand kilocalories per mole. Therefore, given the stronger structural plausibility of the second model, the bridge transformation approach was adopted for all subsequent calculations. This model better reflects the discrete nature of hydration-layer insertion and aligns with observed changes in fibril geometry in Type II collagen. Using the methodology presented above, the total energy contribution of water-mediated bonds is estimated to be 248 kcal/mol in Type I collagen and 72 kcal/mol in Type II collagen (Appendix C).

Interpreting these results requires noting that these energies rest on a set of simplifying assumptions designed to provide a physically grounded representation of water-mediated interactions. Although the estimated number of water bridges per TC molecule does not capture the full extent of the dynamic interactions, it is consistent with structural data and statistical averages, providing a reasonable approximation of their overall energetic contributions. In terms of energy, the adopted 0.46 kcal/mol for single-water bridges is derived from established models and averaged systems, which ensures consistency but may overlook local variations in geometry. Similarly, the $1/r^{3}$ distance-decay model reflects the dipole–dipole nature of these interactions in a uniform medium. The local dielectric heterogeneity is not taken into account. Despite these limitations, the assumptions are physically grounded and provide a coherent estimate of the contribution of water-mediated bonding to fibrillar cohesion under varying hydration conditions.

### VdW interactions

VdW interactions are fundamental components of noncovalent cohesion in protein assemblies in general and in collagen in particular. Although weaker than hydrogen-bonding on a per-contact basis, VdW forces are widespread and additive, acting between all atoms in close proximity. In collagen, these interactions contribute meaningfully to the lateral stabilization between adjacent TC molecules [1,18,19].

To estimate the VdW cohesion energy between adjacent collagen molecules, a computational approach that integrates atomic composition, statistical interaction frequencies, and LJ potential parameters was developed. This method captures the cumulative contribution of the forces by combining the molecular-scale details with the fibril-level organization. It accounts for the types and frequencies of interacting atoms and for their spatial accessibility within the fibril.

The atomic composition of each TC chain based on known residue formulas was first computed by retrieving the full amino acid sequences for Type I and Type II collagen from the UniProt database [30] as detailed in Appendix D. The number of atoms for each atom type was calculated by summing all amino acids while excluding the backbone atoms. For example, 55 arginine (C_6_H_14_N_4_O_2_) residues appear in the α1(I) helix. Each residue contributes a backbone corresponding to the repeating peptide unit –NH–CH–CO–, which contains 2 carbon atoms, 2 oxygen atoms, 1 nitrogen atom, and 4 hydrogen atoms (C_2_H_4_NO_2_). The remaining atoms therefore belong to the side chain. For arginine, this corresponds to a side-chain composition of C_4_H_10_N_3_ after removing the backbone atoms from the full amino acid formula. Therefore, the total arginine side-chain contribution from the 3 α-helices is 165 × (C_4_H_10_N_3_), which corresponds to 660 carbon atoms, 1,650 hydrogen atoms, and 495 nitrogen atoms. These side-chain atom counts were then used to determine the relative frequencies of each atom type used in the interaction probability calculations

Due to the triple-helical nature of the TC molecule, backbone atoms (–N–C_α_–C′–O–H) are deeply buried within the molecular core, where they are involved in extensive intrachain bonding. Their distance from adjacent molecules typically exceeds the range at which VdW interactions are considerable, and they were therefore excluded from the modeling of VdW lateral cohesion. Similarly, side chains that are oriented toward the interior of the triple helix were considered structurally inaccessible for intermolecular contact and were excluded from VdW interaction modeling. Only outward-facing, surface-accessible side chains were retained, as they are geometrically positioned to interact with neighboring TC molecules. Unlike the rigid backbone, these side chains possess geometric flexibility, allowing their reorientation to maintain favorable VdW contact distances, even in hydrated environments where water molecules may occupy the interstitial sites. Based on the symmetry of the molecule, we estimated that half of the side chains are oriented outward (toward neighboring molecules).

Pairwise interaction probabilities were therefore calculated from the relative frequency of each atom type (C, H, O, N, and S) among the accessible atoms. The number of VdW interactions is therefore given by

$$N_{X-Y}=P_{X-Y}*N_{\text{total}}$$ (3)

$$P_{X-Y}=2*f_{X}*f_{Y}$$ (4)

where $f_{X}$ and $f_{Y}$ represent the frequencies of any X and Y elements, respectively; $P_{X-Y}$ represents the probability of occurrence of the bond; and $N_{\text{total}}$ represents the total number of atoms capable of VdW interactions (side-chain atoms). For example, C atoms represent 29.22% of Type I side chains, while O represents 3.95%. The probability of the occurrence of C–O VdW interaction is $\begin{array}{c} P_{C-O}=2*29.22\%*3.95\%=2.31\% \end{array}$, equivalent to a total of 238 VdW interactions. Details of VdW bonds per type, atomic composition, and frequencies are provided in Appendix D.

**Fig. 3.**
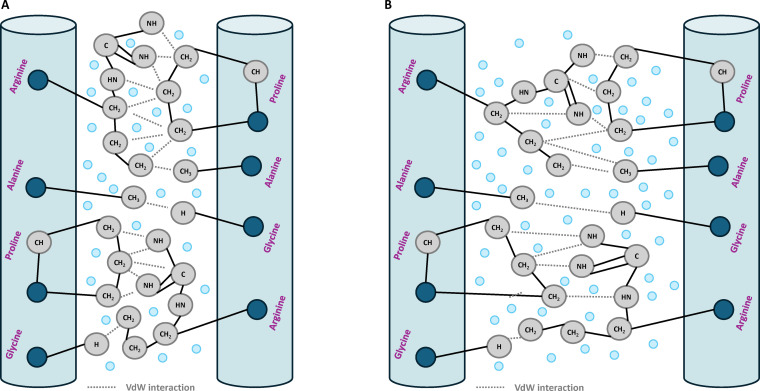
Van der Waals (VdW) interactions in collagen fibrils. (A) Type I collagen: close molecular packing enables numerous short-range VdW contacts between outward-facing side chains (Pro, Ala, Gly, and Arg), strengthening fibrillar cohesion. (B) Type II collagen: greater hydration increases intermolecular spacing and decreases the number and strength of VdW contacts, reducing dispersion-driven cohesion.

For Type I (Fig. 3) collagen, we assumed that the intermolecular spacing remains sufficiently compact to allow outward-facing side chains to reorient and adopt the optimal VdW interaction distances. This reflects the low-hydration, high-density environment typical of tendon-like tissues. Under these conditions, the flexible side chains can maximize contact with neighboring molecules, and all atom–atom distances are assumed at the LJ equilibrium separation. For Type II collagen (Fig. 3), we adopt the same assumption discussed in water-mediated bonds where the central triple-helical core remains rigid, with a fixed diameter of 10.0 Å [41], and where a uniform scaling factor of 1.40 is applied to all side chains’ atom–atom distances, increasing homogeneously all intermolecular distances. Because the attractive component of the LJ potential decays with the inverse sixth power of distance, this geometric expansion leads to a strong attenuation of VdW cohesion energy in Type II fibrils.

The strength of the VdW interactions was calculated by applying a pairwise LJ potential to all atom–atom contacts between accessible side chains. LJ parameters, specifically well depth (*ε*) and atomic separation distance (*σ*), were taken directly from the CHARMM36 force field, which is widely validated for biomolecular simulations [66]. To ensure consistency, representative atom types were selected from the force-field parameter set, which distinguishes atoms of the same element according to their chemical environment. Because each context is associated with different LJ parameters, reflecting variations in VdW radii, only the structurally relevant types were retained. Specifically, the aliphatic carbon type CT2 (sp^3^-hybridized carbon) was chosen to represent outward-facing carbons, as it dominates the side chains of collagen’s abundant residues such as proline, alanine, and hydroxyproline. For oxygen, carbonyl and carboxyl types were selected to capture the dominant polar oxygen environments found in aspartate, glutamate, and hydroxyproline side chains. For nitrogen, the NH1 type (amide nitrogen) was used to represent accessible nitrogen such as those in lysine or arginine residues. Hydrogen atoms were modeled using the HA2 type (Cα-bonded hydrogen), which is the most abundant hydrogen environment in glycine-rich collagen. Self-interaction parameters (C–C, O–O, N–N, and H–H) were taken directly from CHARMM36, while mixed pairs (C–N, O–N, C–O, C–H, O–H, and N–H) were computed using the Lorentz–Berthelot combining rules [67,68]:

$$\sigma_{\mathrm{ij}}=\frac{\sigma_{i}+\sigma_{j}}{2}$$ (5)

$$\varepsilon_{\mathrm{ij}}=\sqrt{\varepsilon_{i}*\varepsilon_{j}}$$ (6)

The total VdW cohesion energy was calculated at 414 kcal/mol per TC molecule in Type I collagen and 125 kcal/mol in Type II (Appendix D). Appendix D provides the detailed calculation procedure, including the statistical estimation of contact frequencies, the number of VdW interaction pairs, and the LJ parameters used to compute the interaction energies. This sharp difference reflects the influence of fibrillar packing density in Type I supporting stronger interactions than the expanded structure of Type II.

These values should be interpreted in light of several modeling considerations. The VdW energy estimates are based on CHARMM36 parameters, optimized for low-dielectric, protein environments, that might not reflect well the solvent effects. As such, dispersion energies may be overestimated in Type II collagen. Another important limitation arises from the assumption that all outward-facing side chains are geometrically flexible enough to reach the optimal LJ equilibrium distance. While this simplification facilitates energy estimation, it does not fully reflect the constraints imposed by the collagen fibril geometry. Only a subset of surface atoms can adopt the ideal LJ contact distance simultaneously, as local packing and side-chain orientations restrict complete optimization. Therefore, the total VdW cohesion energy calculated here are likely overestimated.

### Hydrophobic interaction energy

**Fig. 4.**
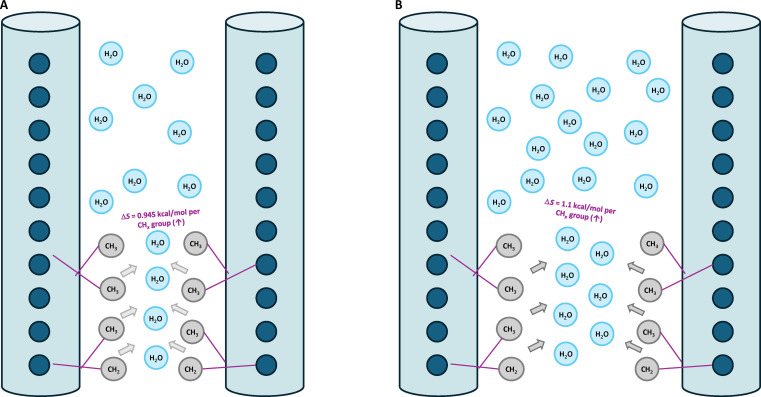
Hydrophobic association between adjacent collagen molecules. (A) Type I collagen: lower hydration results in fewer structured water molecules and a smaller entropic gain (Δ*S* ~ 0.95 kcal/mol per CH*_x_* group). (B) Type II collagen: higher hydration increases the number of ordered water molecules surrounding nonpolar CH_2_/CH_3_ side chains and produces a larger entropic gain (Δ*S* ~ 1.1 kcal/mol per CH*_x_* group).

Hydrophobic interactions are entropically driven associations that arise when nonpolar side chains in molecules aggregate in aqueous environments. In collagen, when hydrophobic groups are surrounded by structured hydration shells as shown in Fig. 4, these ordered water molecules are displaced, resulting in an entropic gain that drives molecular association as described by Dill [27]. Although hydrophobic interactions do not involve specific bonding between side chains, their ability to promote close packing of aligned collagen triple helices adds considerably to the cohesive energy of the fibril. Thermodynamic analyses by Dill [27] established that hydrophobic interactions play an important role in stabilizing protein assemblies, supporting the hypothesis that such interactions contribute meaningfully to the lateral cohesion of collagen fibrils. Notably, the strength of hydrophobic interactions is modulated by the degree of hydration: a greater solvent content enhances water structuring and thus increases the entropic benefit of hydrophobic clustering. Accordingly, hydrophobic interactions are expected to contribute more substantially to fibril cohesion in highly hydrated Type II collagen than in relatively dehydrated Type I collagen tissue.

To quantify the contribution of hydrophobic interactions to the cohesion of Type II collagen fibrils, we focused on nonpolar side chains capable of displacing structured water upon intermolecular contact. The thermodynamic basis follows Pace et al.’s formulation [69], in which each buried CH_2_ or CH_3_ group contributes approximately 1.1 kcal/mol of entropic stabilization in a fully hydrated system (which is representative of Type II collagen). Only CH_2_ and CH_3_ groups were included as hydrophobic contributors, as experimental studies have demonstrated that they predominantly account for the hydrophobic effect [70]. Single-hydrogen CH groups (α-C–H) were excluded because they provide negligible stabilization upon burial [69,71]. Using the amino acid composition of Type II collagen, we identified 1,683 side-chain hydrophobic residues (containing CH_2_ or CH_3_ groups), with a total of 4,152 CH_2_/CH_3_ groups. Detailed residue counts are provided in Appendix E. Because of the triple-helical structure, we assume that half of the side chains are buried inside the triple helix and inaccessible to solvent. Therefore, the total hydrophobic contribution is given by

$$E_{\mathrm{hyd}\left(\mathrm{II}\right)}=\frac{n_{CH_{x}\left(\mathrm{II}\right)}*E_{{\mathrm{CH}}_{x}\left(\mathrm{II}\right)}}{2*\mathrm{So}l_{\text{accessibility}}}=\frac{4,152*1.1}{2*2}=1,142\;\text{kcal}/\mathrm{mol}$$ (7)

where $n_{CH_{x}\left(\mathrm{II}\right)}$ represents the number of CH_2_/CH_3_ bonds in side chains, $E_{{\mathrm{CH}}_{x}\left(\mathrm{II}\right)}$ represents their entropic energy, and the denominator accounts for both the limited solvent accessibility (factor of 2) and the double counting of shared intermolecular contacts (second factor of 2). All hydrophobic side chains not buried within the triple helix were assumed to contribute to hydrophobic energy. This assumption reflects the structural flexibility of collagen fibrils, where side chains are free to reorient and engage in lateral interactions. For consistency, we apply the same assumption to Type I collagen, namely, that half of the CH_2_/CH_3_ side-chain groups are accessible and available for hydrophobic association.

The energy contribution of each group, however, depends not only on the proximity but also on the number of displaced water molecules. In Type II collagen, which resides in a highly hydrated matrix, Pace et al. [69] calculated an energy contribution of 1.1 kcal/mol for each buried CH_2_/CH_3_ group. Based on the established hydration level of 2.4 g of H_2_O/g of dry collagen, we calculated that each residue displaces about 19.2 water molecules (Appendix D). This yields an average entropic contribution of ~0.057 kcal/mol per displaced water molecule. Given the lower hydration value for Type I collagen of 1.6 g of H_2_O/g of dry collagen (corresponding to about 16.5 water molecules per residue), we obtained an energy of $E_{{\mathrm{CH}}_{x}\left(I\right)}=0.057\times 16.5\approx 0.945\;\text{kcal}/\mathrm{mol}$ per CH_2_/CH_3_ group.

Based on the number of hydrophobic groups and the energies presented above, the total hydrophobic interaction energy in Type I collagen was calculated to be 726 kcal/mol versus 1,142 kcal/mol in Type II collagen.

While the above estimates provide a first-order approximation of hydrophobic contributions, several limitations must be noted. First, the use of a fixed per-group energy from Pace et al.’s formulation assumes a fully hydrated environment and may not fully capture local conformational effects within collagen fibrils. Second, the assumption that half of the CH_2_/CH_3_ groups are solvent accessible is a structural simplification, as the actual accessibility depends on local packing and fibril twist. Third, hydration levels used to scale the entropic contribution are based on tissue averages (2.4 g/g for Type II and 1.6 g/g for Type I), which may locally differ inside the microfibril. Collectively, these assumptions may lead to an over or underestimation of the absolute energies. Despite these simplifications, the approach provides a consistent and physically meaningful framework for comparing hydrophobic cohesion between collagen types.

### Coulombic interaction energy

To estimate the attractive electrostatic contribution between charged residues within Type I and Type II collagen fibrils, we considered the interactions arising from side-chain groups that carry a net electrical charge.

Coulombic interactions in collagen fibrils can be broadly divided into long-range electrostatic attractions and short-range, high-strength contacts. The former are strongly attenuated by dielectric screening in the fibrillar environment [72,73] and are omitted here. Instead, we focus on the short-range coulombic interactions, commonly referred to as salt bridges [74], which occur when oppositely charged side chains form direct contacts. These interactions typically involve atom-to-atom distances of 2.8 to 3.2 Å, such as in arginine–aspartate or lysine–glutamate pairs [74,75]. Amino acid residues in a polypeptide chain are linked through peptide bonds, which form neutral amide groups and therefore do not carry formal backbone charges. Accordingly, only residues with formally charged side chains were considered in the coulombic interaction analysis. Therefore, we restricted our analysis to the 4 principal charged residues known to participate in fibril-level stabilization: aspartate (Asp, –COO^−^, −1), glutamate (Glu, –COO^−^, −1), lysine (Lys, –NH_3_^+^, +1), and arginine (Arg, –C(NH_2_)_2_^+^, +1).

Rather than distributing partial charges across multiple atoms within the side chains, each residue was treated as a single electrostatic center carrying its full net charge (±1e) located at the terminal atom of its charged group. This simplification reflects the fact that the charge is mainly concentrated near the end of the side chain and that, at the short salt-bridge distances involved, the entire group behaves as one charge site. Each charged side chain was therefore represented as a unit point charge as parameterized in standard biomolecular force fields [76–78]. All other amino acids are neutral, except for histidine, which may carry a partial positive charge. However, it was excluded due to its low abundance in collagen. These 4 charged side chains in collagen extend both inward and outward from the triple helix and contribute considerably to interfibrillar stabilization. For instance, the arginine side chain (–(CH_2_)_3_–C(NH_2_)_2_^+^), composed of 3 methylene groups and a positively charged group at the end, extends ~6.3 Å from the backbone Cα atom to its terminal carbon [79], based on standard bond lengths and projected geometry [77]. Similarly, the lysine side chain (–(CH_2_)_4_–NH_3_^+^) extends ~6.2 Å, the aspartate side chain (–CH_2_–COO^−^) extends ~3.6 Å, and glutamate (–CH_2_–CH_2_–COO^−^) reaches ~4.8 Å. To evaluate whether these groups can physically interact across fibrillar interfaces, we compared their side-chain extensions with the intermolecular spacing in collagen fibrils.

**Fig. 5.**
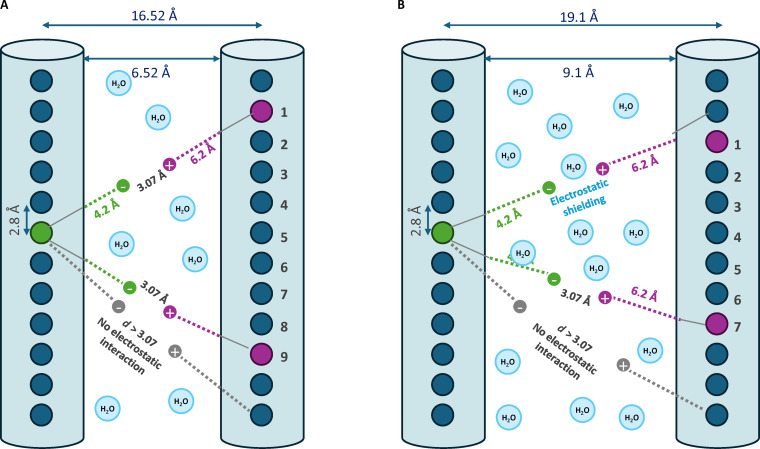
Coulombic interaction configuration between adjacent collagen molecules. (A) Type I collagen: a closer molecular spacing (~16.52 Å) allows oppositely charged side chains to interact across an average separation of ~6.52 Å, spanning 9 potential electrostatic interaction sites. (B) Type II collagen: higher hydration increases intermolecular spacing (~19.1 Å), reducing the strength and the likelihood of effective coulombic pairing to 7 interaction sites.

As established in the hydrogen-bonding analysis, Type I collagen can be approximated as a cylindrical core 10 Å in diameter, with an interatomic distance of 16.52 Å [19], leading to a radial gap of ~6.52 Å (Fig. 5). This gap can easily be spanned by the charged side chains extending between 3.6 and 6.3 Å from each side of the backbone. Along the fibril axis, each residue advances 2.86 Å [1], allowing each charged residue to potentially align with 9 consecutive residues in the neighboring molecule (Appendix E). Given a charged residue frequency of ~8.1% (Appendix E), the probability that at least 1 of the 9 facing residues carries an opposite charge is

$$P_{\text{coul}\left(I\right)}=1-{\left(1-P_{I}\right)}^{n1}=79.5\%$$ (8)

where $P_{I}=16.1\%$ is the ratio of charged terminals and $n_{1}=9$ is the number of terminals from adjacent molecule geometrically accessible. The number of salt bridges $N_{\text{coul}\left(I\right)}$ is therefore given by

$$\begin{array}{c} N_{\text{coul}\left(I\right)}=\\ P_{\text{coul}\left(I\right)}*\frac{N_{\text{charged}\left(I\right)}}{2}=79.5\%*\frac{522}{2}=207\;\text{salt bridges} \end{array}$$ (9)

where $N_{\text{charged}\left(I\right)}=522$ is the number of amino acids charged in Type I collagen.

For Type II collagen, the larger molecular diameter (calculated at ~19.1 Å) yields an interfibrillar gap of ~9.1 Å, which can still be spanned by charged side chains. However, each charged residue in this configuration can potentially align with 7 consecutive residues in the neighboring molecule ($n_{2}=7$). Given the slightly lower charged residue frequency $P_{\mathrm{II}}=15.1\%$ in Type II, the probability that at least 1 of the 7 facing residues carries an opposite charge is calculated as $P_{\text{coul}\left(I\right)}=68.3\%$, and the total number of salt bridges is therefore calculated as 188 bridges.

To evaluate their energetic importance, the interaction strength of these salt bridges was quantified based on the well-established electrostatic behavior of oppositely charged amino acid side chains. These same interactions were extensively characterized by Nayek et al. [80] using Poisson–Boltzmann continuum electrostatics across a wide range of protein structures. In their study, the authors analyzed over 100 salt bridges and reported interaction energies typically ranging from 1.5 to 4.5 kcal/mol, depending on the spatial orientation and local dielectric environment, with a most frequent value of ~3.0 kcal/mol, particularly for geometries involving short-range, atom-to-atom distances of 2.8 to 3.2 Å. This representative energy corresponds to a moderately hydrated protein, which closely resembles the environment within Type I collagen fibrils. Accordingly, 3.0 kcal/mol was adopted as a representative per-pair interaction energy for Type I collagen in this study.

Since Type I and Type II exhibit different hydration levels, their effective dielectric constant *ε* are expected to differ as well. Because coulombic energy is inversely proportional to the local dielectric constant, the strength of the salt bridges in Type II collagen is reduced relative to that in Type I. To quantify this effect, the per-bridge energy in Type II fibrils was scaled according to the ratio of the effective dielectric constants of the 2 systems:

$$E_{\text{coul}}^{\mathrm{II}}=E_{\text{coul}}^{\mathrm{II}}*\frac{\varepsilon^{I}}{\varepsilon^{\mathrm{II}}}$$ (10)

Several approaches have been proposed to estimate the dielectric constant ε in heterogeneous hydrated proteins, including empirical mixture laws [81], Bruggeman effective-medium theory [82], and the Maxwell Garnett approximation [83]. The Maxwell Garnett model was selected because it treats the protein as the continuous phase and water as discrete inclusions, which closely reflects the structural organization of collagen fibrils. Therefore, the effective dielectric constant can be expressed as

$$\varepsilon =\varepsilon_{p}*\frac{\varepsilon_{w}+2\varepsilon_{p}+2\phi_{w}\left(\varepsilon_{w}-\varepsilon_{p}\right)}{\varepsilon_{w}+2\varepsilon_{p}-\phi_{w}\left(\varepsilon_{w}-\varepsilon_{p}\right)}$$ (11)

where $\varepsilon_{w}=78$ is the dielectric constant of water [84], $\varepsilon_{p}=4$ is the dielectric constant of protein [85], and $\phi_{w}$ is the water volume fraction within the fibril.

Using $\phi_{w}^{I}=68.4\%$ for Type I and $\phi_{w}^{\mathrm{II}}$ = 76.3% for Type II (Appendix A), we obtain the dielectric constants $\varepsilon^{I}=21.2$ and $\varepsilon^{\mathrm{II}}=26.9$. This yields a per-interaction energy of 2.37 kcal/mol.

By combining the representative per-pair interaction energy with the number of charged residues, the total coulombic interaction energy is given by

$$E_{\text{coul}\left(I\right)}=N_{\text{coul}\left(I\right)}*e_{\mathrm{cou}\left(I\right)}=207*3=622\;\text{kcal}/\mathrm{mol}$$ (12)

$$E_{\text{coul}\left(\mathrm{II}\right)}=N_{\text{coul}\left(\mathrm{II}\right)}*e_{\text{coul}\left(\mathrm{II}\right)}=188*2.37=443\;\text{kcal}/\mathrm{mol}$$ (13)

While the present approach offers a physically grounded estimate of short-range electrostatic stabilization in collagen fibrils, several assumptions could affect the quantitative precision of the resulting energy values. First, the use of fixed partial charges from standard biomolecular force fields assumes idealized dielectric behavior and does not account for local fluctuations in polarity, potentially introducing deviations from actual interaction energies. Second, the coulombic energy was calculated at a single, fixed interaction distance (3.07 Å) based on VdW radii, whereas actual salt-bridge distances may slightly vary depending on local geometry and flexibility. Third, the estimated number of accessible residues per charged site, based on geometric overlap across fibrils, may be an upper bound, as it accounts for neither the helical twist of the collagen molecule nor the water molecules that might limit the flexibility of the side chains movements, especially in Type II collagen. Repulsive electrostatic interactions between like-charged residues were also neglected in this analysis. Although such interactions are largely screened in the fibrillar environment, the high rotational flexibility of charged side chains allows local reorientation that minimizes same-charge repulsion. As a result, these repulsive forces rarely persist long enough to contribute considerably to the net electrostatic energy, and their exclusion introduces only a minor overestimation of total stabilization. Additionally, contributions from weaker electrostatic effects, such as those involving histidine or dipolar residues, were excluded, which may slightly underestimate the total interaction energy in hydrated environments. Lastly, the charge centers were placed at the terminal atoms of the charged side chains rather than at the centroid of the charged groups. While this simplification slightly shifts the electrostatic reference point, the effect is small [72]. Overall, the proposed method provides a robust and physically consistent estimate of salt-bridge contributions to fibrillar cohesion.

## Discussion

This work presents a mesoscopic model that quantifies the total cohesive energy between collagen molecules in Type I and Type II fibrils, offering a physically grounded approach for understanding the mechanical stiffness of cartilage at the molecular scale. Unlike earlier coarse-grained models that treated fibril cohesion as a single averaged potential [17,18], the present approach decomposes the cohesive energy into hydrogen-bonding, VdW, hydrophobic, water-mediated, and electrostatic components. The model reveals how each interaction is modulated by hydration and packing density, offering new insights into fibril-level mechanics.

One of the key findings of this work is that hydration notably weakens the lateral cohesion between collagen molecules, primarily through an increase in intermolecular spacing. The expansion of the molecular lattice limits the number of close contacts that can form between adjacent TC molecules, thereby reducing both the strength and number of direct hydrogen bonds, VdW interactions, and electrostatic salt bridges. In parallel, the presence of water facilitates the formation of transient water-mediated bridges and enhances hydrophobic exclusion. Altogether, these effects indicate that hydration alters the molecular organization of the fibril, shifting its cohesive balance from strong direct interactions to weaker, solvent-modulated ones.

**Fig. 6.**
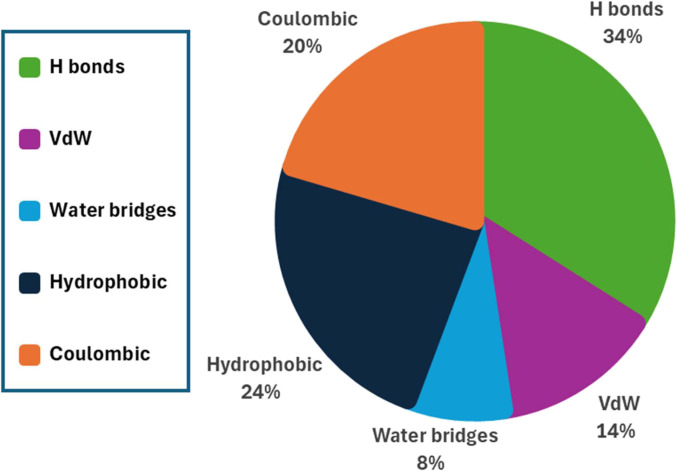
Distribution of intermolecular energy contributions in Type I collagen fibrils. The pie chart illustrates the contributions of hydrogen bonds (34%), van der Waals forces (14%), water-mediated bridges (8%), hydrophobic interactions (24%), and coulombic interactions (20%) to the total cohesive energy.

**Fig. 7.**
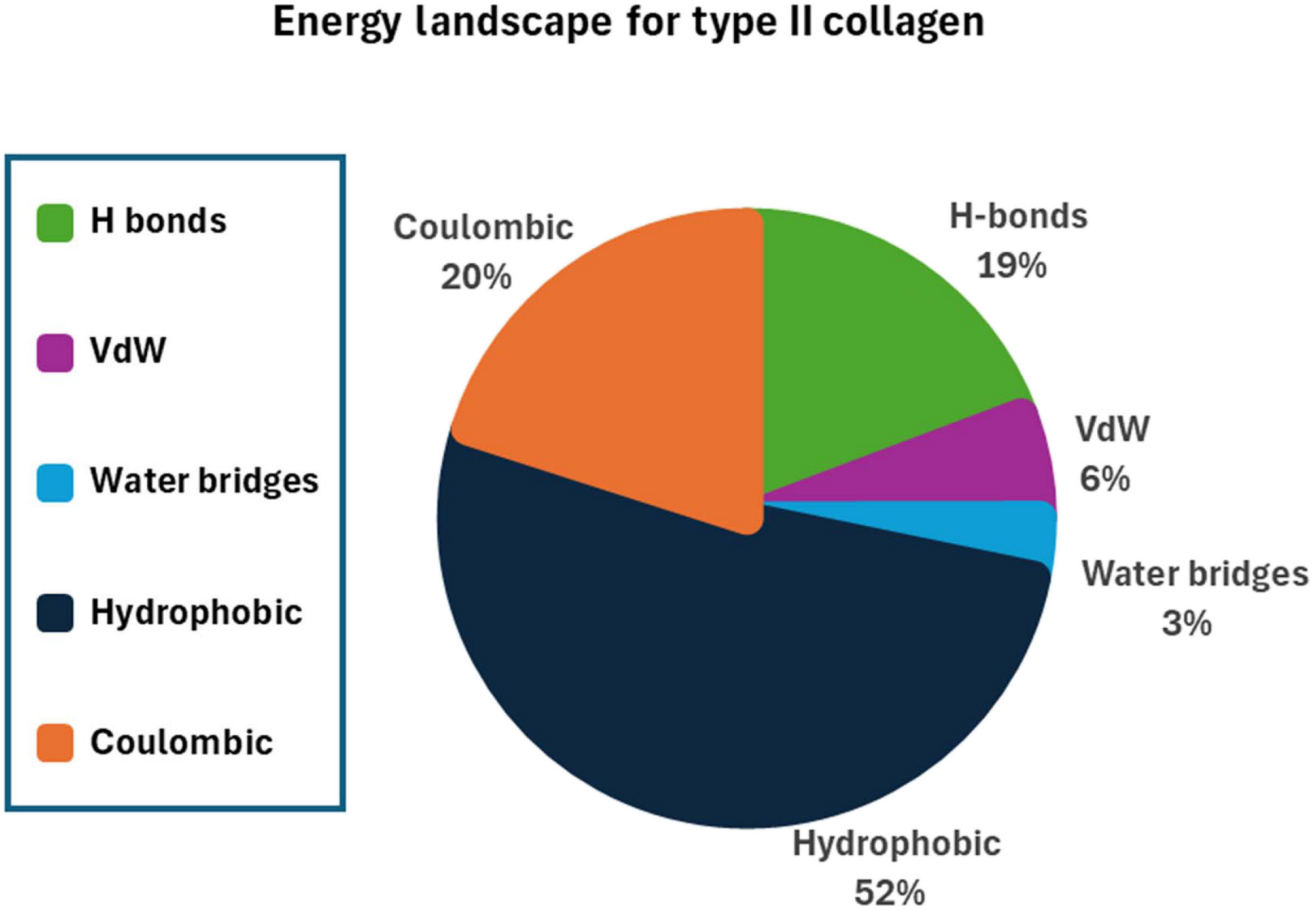
Distribution of intermolecular energy contributions in Type II collagen fibrils. The chart illustrates the contributions of hydrogen bonding (19%), van der Waals interactions (6%), water-mediated bridges (3%), hydrophobic associations (52%), and coulombic interactions (20%) to the total cohesive energy.

**Fig. 8.**
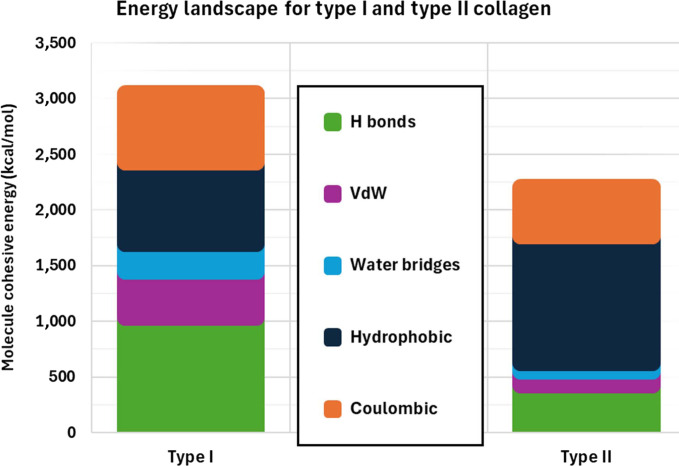
Comparison of cohesive energy components in Type I and Type II collagen fibrils. Type I shows higher total cohesion (3,046 kcal/mol), whereas Type II exhibits lower cohesion (~2,208 kcal/mol) dominated by hydrophobic forces.

The distribution of intermolecular energy contributions in Type I and Type II collagen fibrils is shown in Figs. 6 to 8. Across all 5 interaction categories, hydrophobic interactions emerge as the dominant contributor to cohesion in Type II collagen, accounting for ~1,142 kcal/mol per TC molecule. This aligns with the theory showing that hydrophobic forces contribute substantially to the energetic balance of hydrated protein systems [27,86–88]. By contrast, direct hydrogen-bonding (~426 kcal/mol) and electrostatic interactions (~443 kcal/mol) play secondary but still important roles, with their effectiveness reduced by hydration-induced spacing and dielectric effects. VdW forces (~228 kcal/mol) and water-mediated hydrogen bonds (~72 kcal/mol) contribute the least, consistent with expanded fibrillar geometry and distance-based energy decay.

Compared to Type I collagen, which exhibits ~3,046 kcal/mol of total cohesive energy per molecule, Type II fibrils show a ~27% reduction (~2,208 kcal/mol). This lower cohesion reflects a structural adaptation that enables cartilage to deform under compressive loads and better redistribute stresses, rather than resist uniaxial tension as tendon does. The reduced energy profile also implies that Type II fibrils may be more sensitive to external perturbations. These perturbations can weaken Type II collagen in several ways. For instance, osmotic stress changes the amount of water inside cartilage [89]. When water moves in or out, the space between collagen molecules expands or shrinks, which can loosen the bonds that hold the fibril together. Since Type II fibrils possess inherently greater intermolecular spacing, even small changes in hydration cause a noticeable drop in the number and strength of cohesive contacts between molecules. Enzymatic degradation further reduces stability because matrix metalloproteinases can more easily reach and cut the exposed regions of Type II molecules [90], while the denser Type I fibrils provide less access. Additionally, cross-link disruption removes the covalent bridges that help stabilize the network, allowing molecules to slide past one another [91]. Because Type II collagen naturally contains fewer and weaker cross-links, its structure is more affected by any additional loss. Together, these factors make Type II fibrils more responsive to their environment but also more prone to weakening with aging or disease. Notably, a reduction in tissue hydration can provoke a local compaction response within Type II collagen fibrils, enhancing intermolecular cohesion as reported by Adouni and Dhaher [92]. This phenomenon may be interpreted as an intrinsic autoprotection mechanism.

While the present work focuses on rate-independent cohesive energy, these findings provide a mechanistic basis for interpreting macroscopic viscoelastic parameters. Within a Maxwell-type framework, the lower cohesive energy calculated for Type II collagen results in a reduced activation barrier for intermolecular sliding. This mechanistically contributes to the enhanced viscous dissipation and faster stress relaxation observed in Type II-rich tissues, such as articular cartilage. Furthermore, the combination of reduced cohesion and increased hydration-induced lattice expansion suggests a lower effective surface energy for Type II fibrils, aligning with the requirement for low-friction interfaces in the cartilage matrix.

To place these energetic estimates in context, the cohesive energy magnitudes predicted by this model fall slightly below those implied by Buehler’s coarse-grained framework [18]. This difference is expected because his model did not explicitly account for solvent effects or dielectric screening. Buehler’s framework [18] defined the pairwise potential between coarse-grained beads using LJ parameters ε=6.72kcal/mol and σ=1.34nm but did not report the total energy per TC molecule. When these parameters are combined with the used hexagonal structure and the fibrillar geometry of 218 beads per molecule, the total energy is calculated as

$$E_{\text{buehler}\left(I\right)}=\frac{\varepsilon *N_{\text{beads}}*6*F_{\mathrm{gap}}}{2}=3,911\;\text{kcal}/\mathrm{mol}$$ (14)

This estimate agrees with the 3,046 kcal/mol computed in the present work for Type I collagen, confirming the quantitative consistency of the present model with the energy scale implied by Buehler’s parameters.

By establishing this quantitative consistency with the energy scales of established Type I models, the present work effectively provides a foundational parametric framework for Type II collagen. This addresses an important data gap in the literature, providing the same methodological parity for cartilaginous tissues that the foundational work of Buehler [18] established for tendon and bone. These results offer a set of operational parameters that can be directly integrated into higher-order computational frameworks. Specifically, the cohesive energy densities and the decomposed interaction terms provide the necessary potential energy functions to parameterize coarse-grained MD. Much like the Type I models enabled breakthroughs in studying enzymatic degradation and bone mineralization, these Type II-specific parameters open the gates for simulating cartilage-specific pathologies. Researchers can now move beyond generic protein approximations to analyze how factors such as increased swelling, mineral deposition, or alterations in cross-linking density directly modulate the molecular-scale mechanical integrity of the Type II network.

To our knowledge, no direct experimental measurements have quantified the relative decrease in intermolecular cohesion between Type I and Type II collagen fibrils under comparable conditions. However, mechanical testing at the tissue scale reveals a consistent trend toward reduced strength and stiffness in Type II-rich matrices. Tendons composed predominantly of Type I collagen display tensile moduli of approximately 0.5 to 1.2 GPa and ultimate tensile strengths of 50 to 100 MPa [93,94], whereas articular cartilage, rich in Type II collagen, exhibits tensile moduli of 5 to 20 MPa and strengths of 9 to 40 MPa [95–97], roughly 2-fold lower in stiffness and 2 to 6 times lower in strength. The present reduction in fibrillar cohesive energy thus represents only the molecular-scale component of a broader multiscale weakening. Additional structural factors, including reduced covalent cross-link density, increased molecular disorder, and the random orientation of fibrils within the cartilage matrix, further decrease the effective mechanical strength of Type II-based tissues [98,99]. Overall, these factors explain why cartilage behaves as a soft and load-distributing tissue compared to high-tensile structures such as tendon.

Independent of these tissue-scale comparisons, it is also important to recognize the broader assumptions that define this model. Although the detailed limitations were discussed in earlier sections, a few general points are worth emphasizing. The model assumes uniform hydration and average molecular geometry, without accounting for local variations, dynamic rearrangements, or progressive remodeling. It also focuses on lateral cohesion between molecules, leaving out longitudinal coupling and large-scale fibrillar interactions that can influence tissue mechanics. Even with these simplifications, the model captures the key physical factors governing collagen cohesion and provides a realistic foundation for future refinement and experimental validation. Despite these simplifications, the model offers a robust estimate of the energetic profile of hydrated Type II collagen fibrils. Its modular structure allows for the incorporation of additional effects such as aging-related cross-linking, cartilage degeneration, or scaffold remodeling in tissue engineering applications.

Future developments will extend this framework to include the modeling of longitudinal interactions within the TC molecule, using the same energy-decomposition strategy applied for lateral cohesion. Incorporating these axial forces will enable a more complete characterization of collagenous fibrils, capturing their full 3-dimensional mechanical response. This work therefore represents an initial step toward a comprehensive description of the physical, chemical, and biological processes that govern cohesion within the collagen fibril. By establishing a quantitative basis for bridging molecular-scale energetics with fibrillar-scale mechanics, the present model opens the way for a deeper understanding of how structural organization and biochemical events jointly determine tissue function and degeneration.

## Conclusion

In summary, this study developed a mesoscale model that quantifies the cohesive energy between collagen molecules in Type II fibrils and compares it with its Type I counterpart. By decomposing the total energy into hydrogen-bonding, VdW, hydrophobic, water-mediated, and electrostatic contributions, the model provides the first full energetic map of hydrated Type II collagen at the molecular level.

Quantitatively, Type II collagen exhibits a total cohesive energy of 2,208 kcal/mol per TC molecule, nearly 27% lower than that of Type I (3,046 kcal/mol). This reduction results from hydration-induced lattice expansion from 16.52 to 19.1 Å, which mainly limits direct hydrogen-bond, VdW, and salt-bridge contacts. Among all contributors, hydrophobic interactions dominate in Type II (~52% of total cohesion), followed by coulombic and hydrogen-bonding energies. The weaker VdW and water-mediated terms reflect the expanded molecular spacing and dielectric screening in the hydrated tissue. The model therefore establishes a physical link between hydration, molecular organization, and macroscopic tissue mechanics, clarifying how variations in water content tune intermolecular cohesive energy, which serves as a molecular basis of macroscopic stiffness.

While the present analysis assumes uniform hydration and average molecular geometry, future work should incorporate site-specific enzymatic and nonenzymatic cross-links, which enables predictive simulations of collagenous tissue and therefore a more comprehensive understanding of collagen behavior with aging and diseases.

## Data Availability

All data needed to evaluate the conclusions of this study are present in the paper and in the Supplementary Materials.

## Supplementary Materials

Supplementary 1 Appendices A to F Tables A1 to A3, B1, D1 to D5, E1 and E2, and F1
