# Supplementary material for: How Hydration Weakens Collagen: A Mesoscale Energy Decomposition of Type I and Type II Fibrils
Source: Comput Struct Biotechnol J. 2026 Apr 21;35(1):0050. doi: 10.34133/csbj.0050 (PMC13096680; doi:10.34133/csbj.0050)
Supplement: Supplementary 1 — Appendices A to F Tables A1 to A3, B1, D1 to D5, E1 and E2, and F1 [file csbj.0050.f1.docx]

# Appendix A: Hydrogen-bond energy

1. **Water content in Type I and Type II collagen**

- Water content in Type I collagen

The weight and volume water percentages in Type I collagen are given by:

| $WP_{I\left( W \right)}=\frac{WC_{I}}{1+WC_{I}}=\frac{1.6}{1+1.6}=61.5\%$ | (A.1) |
| --- | --- |
| $WP_{I\left( V \right)}=\frac{\frac{WC_{I}}{{}_{w}}}{\frac{WC_{I}}{{}_{w}}+\frac{1}{{}_{col}}}=\frac{\frac{1.6}{1}}{\frac{1.6}{1}+\frac{1}{1.35}}=68.4\%$ | (A.2) |

Where $WC_{I}$ = 1.6 g H₂O/g dry collagen is the water content in Type I collagen, ${}_{w}=1\frac{g}{cm^{3}}$ is the water density, and ${}_{col}=1.35\frac{g}{cm^{3}}$ is the collagen density (31).

The water volume and collagen dry volume for Type I collagen can therefore be extracted:

| $V_{coll_{I}}=*\frac{D_{coll_{I}}^{2}}{4}*1Å=*\frac{{16.52}^{2}}{4}=214,3 Å^{3}$ | (A.3) |
| --- | --- |
| $WV_{I}=WP_{I\left( V \right)}*V_{Coll_{I}}*1Å=68.4\%* 214,3 Å^{3}= 146,5 Å^{3}$ | (A.4) |
| $CV_{I}=V_{coll_{I}}-WV_{I} = 67,8Å^{3}$ | (A.5) |

Where $V_{coll_{I}}$ is the volume of a single molecule per unit length. $D_{coll_{I}}=16.52 Å$ is the collagen fibril diameter (19)**,** $WV_{I}$is the total water volume per unit length, and $CV_{I}$ is the dry collagen total volume per unit length.

- Water content in Type II collagen

The water content in Type II collagen is calculated as:

| ${WC}_{II}=\frac{{WP}_{II\left( W \right)}}{1-{WP}_{II\left( W \right)}}=\frac{70.5\%}{1-70.5\%}=2.39 g H_{2}O/g Col$ | (A.6) |
| --- | --- |

Where ${WP}_{II\left( W \right)}=70.5\%$ is the water percentage in Type II collagen (34). Consequently, the water volume percentage, the water volume, the total molecule volume, and the molecule diameter are calculated as:

| $WP_{II\left( V \right)}=\frac{\frac{WC_{II}}{{}_{w}}}{\frac{WC_{II}}{{}_{w}}+\frac{1}{{}_{col}}}=\frac{\frac{2.39}{1}}{\frac{2.39}{1}+\frac{1}{1.35}}=76.3\%$ | (A.7) |
| --- | --- |
| ${WV}_{II}=WV_{I}*\frac{{WC}_{II}}{{WC}_{I}}=146,5*\frac{2.39}{1.6}= 218,8 Å^{3}$ | (A.8) |
| $V_{{col}_{II}}= {CV}_{I}+{WV}_{II}=218,8 Å^{3}+67,8 Å^{3}=286,7 Å^{3}$ | (A.9) |
| $D_{{col}_{II}}= \sqrt{\frac{4}{*1Å}V_{{col}_{II}}}=\sqrt{\frac{4}{}286,7}=19.1 Å$ | (A.10) |

Hydration and geometric results are summarized in Table A.1.

Table A.1: Hydration and geometric parameters of Type I and Type II collagen

|  | **Symbol** | **Type I** | **Type II** | **Unit** |
| --- | --- | --- | --- | --- |
| **Molecule diameter** | $D_{col}$ | 16,52 (19) | 19,10 | $Å$ |
| **Volume per unit length** | $V_{col}$ | 214,3 | 286,7 | $Å^{3}$ |
| **Collagen density** | ${}_{col}$ | 1,35 (31) | | $g/\mathrm{cm}^{3}$ |
| **water content** | $WC$ | 1,60 (31) | 2,39 | g H₂O/g col |
| **Water % in weight** | $WP_{\left( W \right)}$ | 61,5% | 70,5% (34) | - |
| **Water % in volume** | $WP_{\left( V \right)}$ | 68,4% | 76,3% | - |
| **Water volume** | $WV$ | 146,5 | 218,8 | $Å^{3}$ |
| **Collagen dry volume** | $CV$ | 67,8 | | $Å^{3}$ |

1. **Number of Hydrogen-bonds**

For Type II collagen, the number of hydrogen-bonds is calculated using Hill’s equation, and assuming Hill’s cooperative index $n=1$ indicating no cooperative binding. Therefore, the Hill’s dissociation constant $K_{d}$ is given by:

| $K_{d}=\frac{C_{I}*(1-{}_{I})}{{}_{I}}=\frac{1.6*(1-92.19\%)}{92.19\%}=0.136$ | (A.11) |
| --- | --- |

Where $C_{I}$ is the volume water fraction in Type I collagen expressed in g H₂O/ g collagen, while ${}_{I}$ is the water bond fraction calculated as

| ${}_{I}=\frac{N-N_{H_{I}}}{N}=\frac{5017-392}{5017}=92.19\%$ | (A.12) |
| --- | --- |

The water bond fraction, the hydrogen-bond fraction, and the total number of hydrogen-bonds in Type II collagen can therefore be calculated as

| ${}_{\mathrm{II}}=\frac{C_{II}^{n}}{K_{d}^{n}+C_{II}^{n}}=\frac{{2.4}^{1}}{{2.4}^{1}+{0.136}^{1}}=94.63\%$ | (A.13) |
| --- | --- |
| $f_{H_{II}}= 1-{}_{\mathrm{II}}=5.37\%$ | (A.14) |
| $N_{H_{II}}=f_{H_{II}}*N=269 bonds$ | (A.15) |

Hydrogen-bond counts are summarized in Table A.2.

Table A.2: Number of hydrogen-bonds in Type I and Type II collagen

|  | **Symbol** | **Type I** | **Type II** | **Unit** |
| --- | --- | --- | --- | --- |
| **Hydrogen-bond fraction** | $f_{H}$ | 7,81% | 5,37% | - |
| **Water-bond fraction** |  | 92,19% | 94,63% | - |
| **Apparent dissociation constant** | $K_{d}$ | 0,136 | | - |
| **Total bonds** | $N$ | 5017 (25) | | - |
| **W-Bond number** | - | 4625 | 4748 | - |
| **Hydrogen-bond number** | $n_{H}$ | 392 (25) | 269 | - |

1. **Hydrogen-bond energy**

The hydrogen-bond energy for Type I collagen is calculated as:

| $E_{H_{I}}=E_{H_{II}}+S*\left( C_{\mathrm{II}}-C_{I} \right)=1.58+1.34*\left( 2.39-1.6 \right)=2.64 kcal/mol$ | (A.16) |
| --- | --- |
| $S=\frac{E_{H_{up}}-E_{H_{II}}}{C_{\mathrm{II}}-0}=\frac{4.79-1.58}{2.39}=1.34\frac{kcal}{mol}(per\frac{g H_{2}O}{g col})$ | (A.17) |

Where S is the calculated slope for energy change with respect to water content, $\mathbf{C}_{\mathbf{I}}$ and $\mathbf{C}_{\mathbf{II}}$ are the water contents in Type I and Type II collagen, and $\boldsymbol{E}_{\boldsymbol{H}_{\boldsymbol{up}}}$ is the dry state hydrogen-bond strength. Table A.3 summarizes hydrogen-bond energy results.

Table A.3: Hydrogen-bond energy for Type I and Type II collagen

|  | **Symbol** | **Type I** | **Type II** | **Unit** |
| --- | --- | --- | --- | --- |
| **Water content** | $C$ | 1.6 (31) | 2.39 | g H₂O/g col |
| **Upper bound energy** | $E_{H_{\mathrm{up}}}$ | 4.79 (40) | | Kcal/mol |
| **Energy slope** | $S$ | 1.34 | | Kcal/mol |
| **Hydrogen-bond energy** | $E_{H}$ | 2.64 | 1.58 (40) | Kcal/mol |

1. **Total hydrogen energy summary**

The total hydrogen energy is therefore given by:

| $E_{H_{I}}=n_{H_{I}}*E_{H_{I}}=1,035 kcal/mol$ | (A.$SEQ Equation \backslash* ARABIC$ $1$) |
| --- | --- |
| $E_{H_{II}}=n_{H_{II}}*E_{H_{II}}=426 kcal/mol$ | (A.$SEQ Equation \backslash* ARABIC$ $2$) |

# Appendix B: Amino acid count in Type I and Type II collagen

The number of amino acids in each chain, as well as in the collagen molecules, was obtained from the UniProt entries (P02452, P08123, and P02458) after trimming the signal peptide, the N- and C-terminal propeptides, and the non-helical telopeptides (37), since these regions do not contribute to the triple-helical structure.

Table B.1: Amino-acid count for type I and Type II collagen

| **Amino Acid** | **α1(I) Count** | **α2 Count** | **α1(II) Count** | **Type I count** | **Type II count** |
| --- | --- | --- | --- | --- | --- |
| Alanine | 123 | 108 | 108 | 354 | 324 |
| Arginine | 55 | 55 | 54 | 165 | 162 |
| Asparagine | 13 | 24 | 12 | 50 | 36 |
| Aspartic Acid | 39 | 25 | 32 | 103 | 96 |
| Glutamic Acid | 50 | 45 | 55 | 145 | 165 |
| Glutamine | 31 | 22 | 17 | 84 | 51 |
| Glycine | 353 | 350 | 351 | 1056 | 1053 |
| Histidine | 3 | 12 | 9 | 18 | 27 |
| Isoleucine | 7 | 18 | 22 | 32 | 66 |
| Leucine | 25 | 34 | 68 | 84 | 204 |
| Lysine | 39 | 31 | 43 | 109 | 129 |
| Methionine | 7 | 5 | 6 | 19 | 18 |
| Phenylalanine | 16 | 12 | 14 | 44 | 42 |
| Proline | 247 | 204 | 296 | 698 | 888 |
| Serine | 41 | 32 | 32 | 114 | 96 |
| Threonine | 19 | 19 | 36 | 57 | 108 |
| Tryptophan | 0 | 0 | 4 | 0 | 12 |
| Tyrosine | 4 | 5 | 14 | 13 | 42 |
| Valine | 24 | 40 | 43 | 88 | 129 |
| **TOTAL** | **1096** | **1041** | **1216** | **3233** | **3648** |

# Appendix C: Water-mediated bridges energy

1. **Number of water-mediated bridges**

Assuming a single water bridge per triplet (36,53), the number of bridges in Type I and Type II collagen is given by

| $n_{{WB}_{I}}=\frac{n_{{amino-acids}_{I}}}{3}=\frac{3233}{3}=1078 bonds$ | (C.1) |
| --- | --- |
| $n_{{WB}_{II}}=\frac{n_{{amino-acids}_{II}}}{3}=\frac{3648}{3}=1216 bonds$ | (C.2) |

Where $n_{{amino-acids}_{I}}$ and $n_{{amino-acids}_{II}}$ are the number of amino acids extracted from the collagen sequence in Appendix 2.

1. **Water-mediated energy (Method 1: Bridge-transformation approach)**

In this approach, hydration-induced lattice expansion converts single-water bridges into double-water bridges. The length of the double water bridges is given by:

| $D_{WB_{II}}= D_{WB_{II}}+D_{W-W}=4.92+2.8 =7.72 Å$ | (C.3) |
| --- | --- |

The bonding energy of a double-water bridges $E_{{WB}_{II}}$ is therefore given by:

| $E_{{WB}_{II}}=\left( \frac{D_{WB_{I}}}{D_{WB_{II}}} \right)^{3}*E_{{WB}_{I}}=\left( \frac{4.92}{7.72} \right)^{3}*0.46=0.119 kcal/mol$ | (C.4) |
| --- | --- |

Where $D_{WB_{I}}$ and $D_{WB_{II}}$ are the bond lengths for the water bridges in Type I and Type II, and $E_{{WB}_{I}}=0.46 kcal/mol$ is the bonding energy of a single-water bridge (48).

Total water mediated energies are therefore given by:

| $E_{{WB}_{I}}=n_{{WB}_{I}}*E_{{WB}_{I}}=248 kcal/mol$ | (C.5) |
| --- | --- |
| $E_{{WB}_{II}}=n_{{WB}_{II}}*E_{{WB}_{II}}=72 kcal/mol$ | (C.6) |

1. **Water-mediated energy (Method 2: Swelling factor Method)**

In this alternative approach, the energy of water-mediated bridges is estimated by applying a swelling factor that accounts for the hydration-induced increase in intermolecular spacing between adjacent tropocollagen molecules.

The Swelling factor is calculated by:

| $S=\frac{D_{II}-D_{core}}{D_{I}-D_{core}}=\frac{9.1}{6.52}=1.396$ | (C.7) |
| --- | --- |

Where $S$ is the calculated swelling factor, $D_{II}=19.1 Å$ is the diameter of type II collagen molecule, $D_{I}=16.52 Å$ is the diameter of Type II collagen molecule diameter, and $D_{core}=10 Å$is the diameter of the triple helix core. The length of the double water bridges is given by:

| $D_{WB_{II}}= D_{WB_{I}}*S=4.92*1.396 =6.87 Å$ | **(C.8)** |
| --- | --- |

The bonding energy of a double-water bridges $E_{{WB}_{II}}$ is therefore given by:

| $E_{{WB}_{II}}=\left( \frac{D_{WB_{I}}}{D_{WB_{II}}} \right)^{3}*E_{{WB}_{I}}=\left( \frac{4.92}{6.87} \right)^{3}*0.46=0.169 kcal/mol$ | **(C.9)** |
| --- | --- |

Where $D_{WB_{I}}$ and $D_{WB_{II}}$ are the bond lengths for the water bridges in Type I and Type II, and $E_{{WB}_{I}}=0.46 kcal/mol$ is the bonding energy of a single-water bridge (48).

Total water mediated energies are therefore given by:

| $E_{{WB}_{I}}=n_{{WB}_{I}}*E_{{WB}_{I}}=248 kcal/mol$ | (C.10) |
| --- | --- |
| $E_{{WB}_{II}}=n_{{WB}_{II}}*E_{{WB}_{II}}=103 kcal/mol$ | (C.11) |

# Appendix D: Van der Waals interaction energy

The atomic decomposition of the side chains participating in VDW interactions in Type I and Type II collagen is presented in the tables below.

Table D.1: Atomic decomposition of the outward facing side chains in Type I Collagen

| **residue** | **Count** | **Chemical Formula** | **Side Chain Atom Count (per Residue)** | | | | **Total Side Chain Atoms (per TC Molecule)** | | | |
| --- | --- | --- | --- | --- | --- | --- | --- | --- | --- | --- |
|  |  |  | **C** | **O** | **N** | **H** | **C** | **O** | **N** | **H** |
| **Alanine** | 354 | C₃H₇NO₂ | 1 | 0 | 0 | 3 | 354 | 0 | 0 | 1062 |
| **Arginine** | 165 | C₆H₁₄N₄O₂ | 4 | 0 | 3 | 10 | 660 | 0 | 495 | 1650 |
| **Asparagine** | 50 | C₄H₈N₂O₃ | 2 | 1 | 1 | 4 | 100 | 50 | 50 | 200 |
| **Aspartic Acid** | 103 | C₄H₇NO₄ | 2 | 2 | 0 | 3 | 206 | 206 | 0 | 309 |
| **Glutamic Acid** | 145 | C₅H₉NO₄ | 3 | 2 | 0 | 5 | 435 | 290 | 0 | 725 |
| **Glutamine** | 84 | C₅H₁₀N₂O₃ | 3 | 1 | 1 | 6 | 252 | 84 | 84 | 504 |
| **Glycine** | 1056 | C₂H₅NO₂ | 0 | 0 | 0 | 1 | 0 | 0 | 0 | 1056 |
| **Histidine** | 18 | C₆H₉N₃O₂ | 4 | 0 | 2 | 5 | 72 | 0 | 36 | 90 |
| **Isoleucine** | 32 | C₆H₁₃NO₂ | 4 | 0 | 0 | 9 | 128 | 0 | 0 | 288 |
| **Leucine** | 84 | C₆H₁₃NO₂ | 4 | 0 | 0 | 9 | 336 | 0 | 0 | 756 |
| **Lysine** | 109 | C₆H₁₄N₂O₂ | 4 | 0 | 1 | 10 | 436 | 0 | 109 | 1090 |
| **Methionine** | 19 | C₅H₁₁NO₂S | 3 | 0 | 0 | 7 | 57 | 0 | 0 | 133 |
| **Phenylalanine** | 44 | C₉H₁₁NO₂ | 7 | 0 | 0 | 7 | 308 | 0 | 0 | 308 |
| **Proline** | 698 | C₅H₉NO₂ | 3 | 0 | 0 | 5 | 2094 | 0 | 0 | 3490 |
| **Serine** | 114 | C₃H₇NO₃ | 1 | 1 | 0 | 3 | 114 | 114 | 0 | 342 |
| **Threonine** | 57 | C₄H₉NO₃ | 2 | 1 | 0 | 5 | 114 | 57 | 0 | 285 |
| **Tyrosine** | 13 | C₉H₁₁NO₃ | 7 | 1 | 0 | 7 | 91 | 13 | 0 | 91 |
| **Valine** | 88 | C₅H₁₁NO₂ | 3 | 0 | 0 | 7 | 264 | 0 | 0 | 616 |
| **TOTAL atoms in side chains** | | | | | | | **6021** | **814** | **774** | **12995** |
| **TOTAL atoms in outward facing side chains** | | | | | | | **3011** | **407** | **387** | **6498** |

Table D.2: Atomic decomposition of the outward facing side chains in Type II Collagen

| **residue** | **Count** | **Chemical Formula** | **Side Chain Atom Count (per Residue)** | | | | **Total Side Chain Atoms (per TC Molecule)** | | | |
| --- | --- | --- | --- | --- | --- | --- | --- | --- | --- | --- |
|  |  |  | **C** | **O** | **N** | **H** | **C** | **O** | **N** | **H** |
| **Alanine** | 324 | C₃H₇NO₂ | 1 | 0 | 0 | 3 | 324 | 0 | 0 | 972 |
| **Arginine** | 162 | C₆H₁₄N₄O₂ | 4 | 0 | 3 | 10 | 648 | 0 | 486 | 1620 |
| **Asparagine** | 36 | C₄H₈N₂O₃ | 2 | 1 | 1 | 4 | 72 | 36 | 36 | 144 |
| **Aspartic acid** | 96 | C₄H₇NO₄ | 2 | 2 | 0 | 3 | 192 | 192 | 0 | 288 |
| **Glutamic acid** | 165 | C₅H₉NO₄ | 3 | 2 | 0 | 5 | 495 | 330 | 0 | 825 |
| **Glutamine** | 51 | C₅H₁₀N₂O₃ | 3 | 1 | 1 | 6 | 153 | 51 | 51 | 306 |
| **Glycine** | 1053 | C₂H₅NO₂ | 0 | 0 | 0 | 1 | 0 | 0 | 0 | 1053 |
| **Histidine** | 27 | C₆H₉N₃O₂ | 4 | 0 | 2 | 5 | 108 | 0 | 54 | 135 |
| **Isoleucine** | 66 | C₆H₁₃NO₂ | 4 | 0 | 0 | 9 | 264 | 0 | 0 | 594 |
| **Leucine** | 204 | C₆H₁₃NO₂ | 4 | 0 | 0 | 9 | 816 | 0 | 0 | 1836 |
| **Lysine** | 129 | C₆H₁₄N₂O₂ | 4 | 0 | 1 | 10 | 516 | 0 | 129 | 1290 |
| **Methionine** | 18 | C₅H₁₁NO₂S | 3 | 0 | 0 | 7 | 54 | 0 | 0 | 126 |
| **Phenylalanine** | 42 | C₉H₁₁NO₂ | 7 | 0 | 0 | 7 | 294 | 0 | 0 | 294 |
| **Proline** | 888 | C₅H₉NO₂ | 3 | 0 | 0 | 5 | 2664 | 0 | 0 | 4440 |
| **Serine** | 96 | C₃H₇NO₃ | 1 | 1 | 0 | 3 | 96 | 96 | 0 | 288 |
| **Threonine** | 108 | C₄H₉NO₃ | 2 | 1 | 0 | 5 | 216 | 108 | 0 | 540 |
| **Tryptophan** | 12 | C₁₁H₁₂N₂O₂ | 9 | 0 | 1 | 8 | 108 | 0 | 12 | 96 |
| **Tyrosine** | 42 | C₉H₁₁NO₃ | 7 | 1 | 0 | 7 | 294 | 42 | 0 | 294 |
| **Valine** | 129 | C₅H₁₁NO₂ | 3 | 0 | 0 | 7 | 387 | 0 | 0 | 903 |
| **TOTAL atoms in side chains** | | | | | | | **7701** | **855** | **768** | **16044** |
| **TOTAL atoms in outward facing side chains** | | | | | | | **3851** | **428** | **384** | **8022** |

The frequency of outward facing atoms is extracted from Table D.1 and Table D.2

Table D.3: Number of outward facing atoms in side chains in Type I and Type II collagen.

| **Atom type** | **Type I** | | **Type II** | |
| --- | --- | --- | --- | --- |
|  | **Number** | **frequency** | **Number** | **Frequency** |
| **C** | 3011 | 29.22% | 3851 | 30.36% |
| **O** | 407 | 3.95% | 428 | 3.37% |
| **N** | 387 | 3.76% | 384 | 3.03% |
| **H** | 6498 | 63.07% | 8022 | 63.25% |
| **Total** | **10302** | **-** | **12684** | **-** |

Using the frequencies presented in Table D.3, the bond frequencies are estimated as follows:

| $P_{X-X}=\left( f_{X} \right)^{2}$ | (D.1) |
| --- | --- |
| $P_{X-Y}=2*f_{X}* f_{Y}$ | (D.2) |
| $N_{X-Y}=P_{X-Y}*N_{total}$ | (D.3) |

Where $f_{X}$, and $f_{Y}$ represent the frequencies of any X and Y elements, $P_{X-Y}$ represents the probability of occurrence of the bond, and $N_{total}$ represents the total number outward facing side chain atoms ( $N_{total}=10302$ for Type I collagen, and $N_{total}=12684$ for Type II collagen )

Table D.4: Estimated number and frequency of VDW interaction in Type I and Type II collagen.

| **Pair** | **Type I** | | **Type II** | |
| --- | --- | --- | --- | --- |
|  | **Frequency** | **number** | **frequency** | **number** |
| **C-C** | 8.54% | 880 | 9.22% | 1169 |
| **O-O** | 0.16% | 16 | 0.11% | 14 |
| **N-N** | 0.14% | 15 | 0.09% | 12 |
| **H-H** | 39.78% | 4098 | 40.00% | 5074 |
| **C-O** | 2.31% | 238 | 2.05% | 260 |
| **C-N** | 2.20% | 226 | 1.84% | 233 |
| **C-H** | 36.86% | 3797 | 38.40% | 4871 |
| **O-N** | 0.30% | 31 | 0.20% | 26 |
| **O-H** | 4.98% | 513 | 4.26% | 541 |
| **N-H** | 4.74% | 488 | 3.83% | 486 |

**Table D.5: Lennard-Jones parameters used to model van der Waals interactions in collagen, based on CHARMM36 atom types.**

| **Pair** | **σ** $\boldsymbol{[}\mathbf{Å]}$ | **ε** $\mathbf{[}\mathbf{Kcal/mol}\mathbf{]}$ | **E_VDW I_** $\mathbf{[}\mathbf{Kcal/mol}\mathbf{]}$ | **E_VDW II_** $\mathbf{[}\mathbf{Kcal/mol}\mathbf{]}$ |
| --- | --- | --- | --- | --- |
| **C-C** | 3.58 | 0.056 | 0.056 | 0.014 |
| **O-O** | 3.03 | 0.120 | 0.120 | 0.030 |
| **N-N** | 3.30 | 0.200 | 0.200 | 0.050 |
| **H-H** | 2.39 | 0.032 | 0.032 | 0.027 |
| **C-O** | 3.31 | 0.082 | 0.082 | 0.021 |
| **C-N** | 3.44 | 0.106 | 0.106 | 0.039 |
| **C-H** | 2.99 | 0.042 | 0.042 | 0.011 |
| **O-N** | 3.17 | 0.155 | 0.155 | 0.016 |
| **O-H** | 2.71 | 0.062 | 0.062 | 0.020 |
| **N-H** | 2.85 | 0.080 | 0.080 | 0.008 |

The atomic decomposition of the side chains participating in VDW interactions in Type I and Type II collagen is extracted from Appendix B and presented in the table below.

1. **Total Van Der Waals energy summary**

The total VSW energy is therefore given by:

| $E_{{VDW}_{I}}=\sum_{bond-types} n_{bonds_{I}}*E_{VDW-bond}=414 kcal/mol$ | (D.4) |
| --- | --- |
| $E_{{VDW}_{II}}=\sum_{bond-types} n_{bonds_{II}}*E_{VDW-bond}=125 kcal/mol$ | (D.5) |

Where the sum over $"bond-types"$ represents the different VDW pairs, ${"n}_{bonds}"$ represents the number of each VDW pair bond given in Table D.4, while ${"E}_{VDW-bond}"$ is the Energy of each VDW bond using the LJ formulation given by -
$E_{VDW-bond}=4*\left( \left( \frac{r_{\mathrm{ij}}}{\sigma_{\mathrm{ij}}} \right)^{12}-\left( \frac{r_{\mathrm{ij}}}{\sigma_{\mathrm{ij}}} \right)^{6} \right)$ and shown in Table D.5

# Appendix E: Coulombic interaction energy

1. **Dielectric constant calculation**

Dielectric constant in type I and Type II collagen are calculated using the Maxwell–Garnett approximation.

| ${}_{I}={}_{p}*\frac{{}_{w}+2{}_{p}+2{}_{wI}\left( {}_{w}-{}_{p} \right)}{{}_{w}+2{}_{p}-{}_{wI}\left( {}_{w}-{}_{p} \right)}=4*\frac{78+2*4+2*0.684 \left( 78-4 \right)}{78+2*4-0.684 \left( 78-4 \right)}=21.14$ | (E.1) |
| --- | --- |
| ${}_{II}={}_{p}*\frac{{}_{w}+2{}_{p}+2{}_{wII}\left( {}_{w}-{}_{p} \right)}{{}_{w}+2{}_{p}-{}_{wII}\left( {}_{w}-{}_{p} \right)}=4*\frac{78+2*4+2*0.763 \left( 78-4 \right)}{78+2*4-0.763 \left( 78-4 \right)}=26.97$ | (E.2) |

Where ${}_{I}$ and ${}_{II}$ are the dielectric constants of Type I and Type II fibrils, ${}_{w}=78$ is the dielectric function of water, ${}_{p}=4$ is the dry protein dielectric constant, ${}_{wI}=0.684$ is the water volume fraction in Type I, and ${}_{wII}=0.763$ is the water volume fraction in Type II.

1. **Per-interaction salt bridge energy**

Since the electric charges are similar in type I and Type II, the ratio of dielectric constants is equal to the energy ratios. Using an energy of $E_{salt_{I}}=3 kcal/mol$(79) for type I, the single interaction energy for type II is obtained.

| $R=\frac{E_{salt_{II}}}{E_{salt_{I}}}=\frac{\frac{q_{1}.q_{2}}{4..{}_{II}.r}}{\frac{q_{1}.q_{2}}{4..{}_{I}.r}}=\frac{{}_{I}}{{}_{II}}=\frac{21.14}{26.97}=0.784$ | (E.3) |
| --- | --- |
| $E_{salt_{I}}=3kcal/mol$ | (E.4) |
| $E_{salt_{II}}= E_{salt_{I}}*R=3*0.784=2.35 kcal/mol$ | (E.5) |

1. **Charge distribution in collagen sequences**

The distribution of charged residues in Type I and Type II collagen sequences is summarized in Table E.1. Only residues carrying formal side-chain charges (Lys, Arg, Asp, and Glu) were considered charged sites in the Coulombic interaction calculation, while all other residues were treated as electrically neutral.

**Table E.1: Charge distribution of amino acids in Type I and Type II collagen.**

| **Charge distribution** | | | |
| --- | --- | --- | --- |
| **Amino Acid** | **Partial charge** | **Type I count** | **Type II count** |
| **Alanine** | 0 | 354 | 324 |
| **Arginine** | 1 | 165 | 162 |
| **Asparagine** | 0 | 50 | 36 |
| **Aspartic Acid** | -1 | 103 | 96 |
| **Glutamic Acid** | -1 | 145 | 165 |
| **Glutamine** | 0 | 84 | 51 |
| **Glycine** | 0 | 1056 | 1053 |
| **Histidine** | 0 | 18 | 27 |
| **Isoleucine** | 0 | 32 | 66 |
| **Leucine** | 0 | 84 | 204 |
| **Lysine** | 1 | 109 | 129 |
| **Methionine** | 0 | 19 | 18 |
| **Phenylalanine** | 0 | 44 | 42 |
| **Proline** | 0 | 698 | 888 |
| **Serine** | 0 | 114 | 96 |
| **Threonine** | 0 | 57 | 108 |
| **Tryptophan** | 0 | 0 | 12 |
| **Tyrosine** | 0 | 13 | 42 |
| **Valine** | 0 | 88 | 129 |
| **TOTAL** | **-** | **3233** | **3648** |
| **TOTAL Charged** | **-** | **522** | **552** |
|  | **-** | **16.15%** | **15.13%** |

1. **Number of potential coulombic interaction sites**

The number of potential sites is calculated by assuming that each charged amino acid side chain is free to span to reach other amino acid side chains from adjacent molecules. The potential length of each possible interaction, as well as the maximum length leading to a possible interaction are calculated as:

| $L_{n}= \sqrt{\left( n*offset \right)^{2}+{(d-{}_{core})}^{2}}$ | (E.7) |
| --- | --- |
| $L_{max}= 2*L_{sideChain}+{}_{c}=2*5.225+3.07=13.52 \mathbf{Å}$ | (E.8) |

Where $L_{sideChain}=5.225 Å$ is the average length of charges side chain lengths, ${}_{c}=3.07 Å$ is the typical short range interaction distance, d is the hexagonal lattice constant, ${}_{core}=10 Å$ is the diameter of the triple helix, $offset=2.8 Å$ represents the distance separating each consecutive amino acids on the triple helix, and $n$ represents the number of amino acid jumps in the longitudinal direction of the molecule.

**Table E.2: Length of potential coulombic interaction side chains lengths.**

| **Amino Acid shift (n)** | $\boldsymbol{L}_{\boldsymbol{n}}$ **(Type I) [**$\mathbf{Å}$**]** | $\boldsymbol{L}_{\boldsymbol{n}}$ (**Type II) [**$\mathbf{Å}$**]** |
| --- | --- | --- |
| **0** | **6,52** | **9,10** |
| **1 & (-1)** | **7,10** | **9,53** |
| **2 & (-2)** | **8,59** | **10,69** |
| **3 & (-3)** | **10,63** | **12,39** |
| **4 & (-4)** | **12,96** | 14,43 |
| **5 & (-5)** | 15,44 | 16,70 |
| $\boldsymbol{L}_{\boldsymbol{max}}$ **[**$\mathbf{Å}$**]** | 13,52 | |

The probability of an interaction occurrence and the total number of charged interactions are therefore given by:

| $P_{coul (I)}= 1-\left( 1-P_{I} \right)^{9}=1-\left( 1-16.15\% \right)^{9}=79.5\%$ | (E.9) |
| --- | --- |
| $P_{coul (II)}= 1-\left( 1-P_{II} \right)^{7}=1-\left( 1-15.13\% \right)^{7}=68.3\%$ | (E.10) |
| $N_{coul (I)}=P_{coul (I)}*\frac{N_{charged\left( I \right)}}{2}= 79.5\%*\frac{522}{2}=207 salt bridges$ | (E.11) |
| $N_{coul (II)}=P_{coul (II)}*\frac{N_{charged\left( II \right)}}{2}= 68.3\%*\frac{552}{2}=188 salt bridges$ | (E.12) |

# Appendix F: Hydrophobic interaction energy

1. **Number of hydrophobic groups**

Hydrophobic interactions are estimated from the number of non-polar side-chain CH₂ and CH₃ groups capable of displacing structured water molecules upon intermolecular contact. The total number of hydrophobic groups is given by:

| $N_{CH_{x}}=\sum_{i} n_{i} . g_{i}$ | (F.1) |
| --- | --- |

Where $n_{i}$ represents number of residues of type $i$, and $g_{i}$represents the number of $CH_{2}$ or $CH_{3}$ groups in residue $i$ obtained from the amino-acid composition given in Appendix B:

Table F.1: Number of outward facing CH_x_ groups in Type I and Type II collagen.

| **Residue** | $\boldsymbol{g}_{\boldsymbol{i}}$ | **Type I** | | **Type II** | |
| --- | --- | --- | --- | --- | --- |
|  |  | $\boldsymbol{n}_{\boldsymbol{i}}$ | $\boldsymbol{n}_{\boldsymbol{i}}\boldsymbol{.}\boldsymbol{g}_{\boldsymbol{i}}$ | $\boldsymbol{n}_{\boldsymbol{i}}$ | $\boldsymbol{n}_{\boldsymbol{i}}\boldsymbol{.}\boldsymbol{g}_{\boldsymbol{i}}$ |
| **proline** | 3 | 698 | 2094 | 888 | 2664 |
| **alanine** | 1 | 354 | 354 | 324 | 324 |
| **leucine** | 3 | 84 | 252 | 204 | 612 |
| **valine** | 2 | 88 | 176 | 129 | 258 |
| **isoleucine** | 3 | 32 | 96 | 66 | 198 |
| **phenylalanine** | 1 | 44 | 44 | 42 | 42 |
| **methionine** | 3 | 19 | 57 | 18 | 54 |
| **Total** | | **1319** | **3073** | **1671** | **4152** |

The total number of $CH_{2}$ and $CH_{3}$ groups is therefore given by:

| $N_{CH_{x}\left( I \right)}=3073$ | (F.2) |
| --- | --- |
| $N_{CH_{x}\left( II \right)}=4152$ | (F.3) |

1. **Effective hydrophobic groups**

Due to the triple-helical geometry of tropocollagen, approximately half of the side chains are oriented toward the interior of the helix and are therefore inaccessible to intermolecular contact. Moreover, since each hydrophobic contact is shared by two interacting molecules, the number of effective interactions becomes:

| $N_{hyd}=\frac{N_{CH_{x}}}{2*2}$ | (F.4) |
| --- | --- |

1. **Hydrophobic interaction energy**

The hydrophobic interaction energy per group corresponds to the entropic stabilization per buried CH₂/CH₃ group and is reported by Pace et al. (70):

| $\varepsilon_{hyd}^{II}=1.1\text{ kcal/mol}$ | (F.5) |
| --- | --- |

Because hydrophobic stabilization depends on the number of displaced water molecules, the energy contribution was scaled based on the ratio of water molecules available per accessible $CH_{x}$ group in the respective fibrils. The number of water molecules per accessible group $CH_{x}$was determined for each type:

| ${NW}_{/CH_{x\left( I \right)}}=\frac{{NW}_{(I)}}{{NCH}_{x(I)}}=\frac{m_{collagen_{I}}*\frac{{WC}_{I}}{m_{H2O}}}{{NCH}_{x(I)}}=\frac{285000*\frac{1.6}{18}}{3073}=16.49$ | (F.6) |
| --- | --- |
| ${NW}_{/CH_{x\left( II \right)}}=\frac{{NW}_{(II)}}{{NCH}_{x(II)}}=\frac{m_{collagen_{II}}*\frac{{WC}_{II}}{m_{H2O}}}{{NCH}_{x(II)}}=\frac{300000*\frac{2.49}{18}}{4152}=19.19$ | (F.7) |
| $\varepsilon_{hyd}^{I}=\varepsilon_{hyd}^{II}\cdot\frac{{NW}_{/CH_{x\left( I \right)}}}{{NW}_{/CH_{x\left( II \right)}}}=1.1\cdot\frac{16.49}{19.19}=0.95\text{ kcal/mol}$ | (F.8) |

Where$m_{collagen_{I}}=285000g/mol$, $m_{collagen_{II}}=300000g/mol$ are the molecular masses of Type I and Type II tropocollagen, respectively. ${WC}_{I}= 1.6\text{ }g\text{ }H_{2}O/g\text{ }collagen$ is, and ${WC}_{II}= 2.39\text{ }g\text{ }H_{2}O/g\text{ }collagen$ are hydration levels obtained in Appendix A. ${NCH}_{x(I)}$ and ${NCH}_{x(II)}$ are the total numbers of accessible CH₂/CH₃ groups in Type I and Type II collagen, respectively, obtained from the amino-acid composition Table F.1.

1. **Total hydrophobic interaction energy**

The total hydrophobic interaction energy per tropocollagen molecule is therefore given by:

| $E_{hyd(I)}=\frac{N_{CH_{x(I)}}}{4}\cdot\varepsilon_{hyd\left( I \right)}=\frac{3073}{4}\cdot0.95=726\text{ kcal/mol}$ | (F.9) |
| --- | --- |
| $E_{hyd(II)}=\frac{N_{CH_{x(II)}}}{4}\cdot\varepsilon_{hyd\left( II \right)}=\frac{4152}{4}\cdot1.1=1142\text{ kcal/mol}$ | (F.10) |

# References

1. Shoulders MD, Raines RT. Collagen Structure and Stability. Annu Rev Biochem. 2009 Jun 1;78(1):929–58.

2. Mienaltowski MJ, Birk DE. Structure, Physiology, and Biochemistry of Collagens. In 2014. p. 5–29.

3. Kadler KE, Baldock C, Bella J, Boot-Handford RP. Collagens at a glance. J Cell Sci. 2007 Jun 15;120(12):1955–8.

4. Fratzl P, Weinkamer R. Nature’s hierarchical materials. Prog Mater Sci. 2007 Nov;52(8):1263–334.

5. Eyre D. Collagen of articular cartilage. Arthritis Res. 2002;4(1):30.

6. Martel-Pelletier J, Boileau C, Pelletier JP, Roughley PJ. Cartilage in normal and osteoarthritis conditions. Best Pract Res Clin Rheumatol. 2008 Apr;22(2):351–84.

7. Adouni M, Alkhatib F, Gouissem A, Faisal TR. Knee joint biomechanics and cartilage damage prediction during landing: A hybrid MD-FE-musculoskeletal modeling. PLoS One. 2023 Aug 3;18(8):e0287479.

8. Sophia Fox AJ, Bedi A, Rodeo SA. The Basic Science of Articular Cartilage: Structure, Composition, and Function. Sports Health: A Multidisciplinary Approach. 2009 Nov 2;1(6):461–8.

9. Bielajew BJ, Hu JC, Athanasiou KA. Collagen: quantification, biomechanics and role of minor subtypes in cartilage. Nat Rev Mater. 2020 Jul 20;5(10):730–47.

10. Jürgensen HJ, Madsen DH, Ingvarsen S, Melander MC, Gårdsvoll H, Patthy L, et al. A Novel Functional Role of Collagen Glycosylation. Journal of Biological Chemistry. 2011 Sep;286(37):32736–48.

11. Mow VC, Ratcliffe A, Robin Poole A. Cartilage and diarthrodial joints as paradigms for hierarchical materials and structures. Biomaterials. 1992;13(2):67–97.

12. Han L, Frank EH, Greene JJ, Lee HY, Hung HHK, Grodzinsky AJ, et al. Time-Dependent Nanomechanics of Cartilage. Biophys J. 2011 Apr;100(7):1846–54.

13. Wenger MPE, Bozec L, Horton MA, Mesquida P. Mechanical Properties of Collagen Fibrils. Biophys J. 2007 Aug;93(4):1255–63.

14. Petitjean N, Canadas P, Royer P, Noël D, Le Floc’h S. Cartilage biomechanics: From the basic facts to the challenges of tissue engineering. J Biomed Mater Res A. 2023 Jul 30;111(7):1067–89.

15. Andriotis OG, Nalbach M, Thurner PJ. Mechanics of isolated individual collagen fibrils. Acta Biomater. 2023 Jun;163:35–49.

16. Buehler MJ. Nanomechanics of collagen fibrils under varying cross-link densities: Atomistic and continuum studies. J Mech Behav Biomed Mater. 2008 Jan;1(1):59–67.

17. Gautieri A, Vesentini S, Redaelli A, Buehler MJ. Hierarchical Structure and Nanomechanics of Collagen Microfibrils from the Atomistic Scale Up. Nano Lett. 2011 Feb 9;11(2):757–66.

18. Buehler MJ. Nature designs tough collagen: Explaining the nanostructure of collagen fibrils. Proceedings of the National Academy of Sciences. 2006 Aug 15;103(33):12285–90.

19. Orgel JPRO, Irving TC, Miller A, Wess TJ. Microfibrillar structure of type I collagen *in situ*. Proceedings of the National Academy of Sciences. 2006 Jun 13;103(24):9001–5.

20. Fu I, Case DA, Baum J. Dynamic Water-Mediated Hydrogen Bonding in a Collagen Model Peptide. Biochemistry. 2015 Oct 6;54(39):6029–37.

21. Gouissem A, Mbarki R, Al Khatib F, Adouni M. Multiscale Characterization of Type I Collagen Fibril Stress–Strain Behavior under Tensile Load: Analytical vs. MD Approaches. Bioengineering. 2022 Apr 28;9(5):193.

22. Gouissem A, Alkhatib F, Adouni M. Investigating the influence of mineral content changes on mechanical properties through ligament insertion. Frontiers in Aging. 2025 Jul 7;6.

23. Kamml J, Acevedo C, Kammer DS. Advanced-Glycation Endproducts: How cross-linking properties affect the collagen fibril behavior. J Mech Behav Biomed Mater. 2023 Dec;148:106198.

24. Adouni M, Gouissem A, Al khatib F, Mbarki R. AGES effect on the biomechanics of the knee tendon. Results in Engineering. 2023 Jun;18:101155.

25. Streeter I, de Leeuw NH. A molecular dynamics study of the interprotein interactions in collagen fibrils. Soft Matter. 2011;7(7):3373.

26. Pálfi VK, Perczel A. Stability of the hydration layer of tropocollagen: A QM study. J Comput Chem. 2010 Mar 30;31(4):764–77.

27. Dill KA. Dominant forces in protein folding. Biochemistry. 1990 Aug 7;29(31):7133–55.

28. Banerjee K, Rasheeda K, Tarannum A, Fathima NN. Structural and mechanical behavior of type-I collagen fibrils in presence of induced electrostatic interactions through ionic liquids. Biophys Chem. 2024 Apr;307:107192.

29. Vassaux M. Heterogeneous Structure and Dynamics of Water in a Hydrated Collagen Microfibril. Biomacromolecules. 2024 Aug 12;25(8):4809–18.

30. Orgel JPRO, Persikov A V., Antipova O. Variation in the Helical Structure of Native Collagen. PLoS One. 2014 Feb 24;9(2):e89519.

31. Cameron IL, Short NJ, Fullerton GD. Verification of simple hydration/dehydration methods to characterize multiple water compartments on Tendon Type 1 Collagen. Cell Biol Int. 2007 Jun 2;31(6):531–9.

32. Fullerton GD, Rahal A. Collagen structure: The molecular source of the tendon magic angle effect. Journal of Magnetic Resonance Imaging. 2007 Feb 26;25(2):345–61.

33. Cederlund AA, Aspden RM. Walking on water: revisiting the role of water in articular cartilage biomechanics in relation to tissue engineering and regenerative medicine. J R Soc Interface. 2022 Aug 3;19(193).

34. Crolla JP, Lawless BM, Cederlund AA, Aspden RM, Espino DM. Analysis of hydration and subchondral bone density on the viscoelastic properties of bovine articular cartilage. BMC Musculoskelet Disord. 2022 Dec 8;23(1):228.

35. Grynpas MD, Eyre DR, Kirschner DA. Collagen type II differs from type I in native molecular packing. Biochimica et Biophysica Acta (BBA) - Protein Structure. 1980 Dec;626(2):346–55.

36. Bella J, Brodsky B, Berman HM. Hydration structure of a collagen peptide. Structure. 1995 Sep;3(9):893–906.

37. Bateman A, Martin MJ, Orchard S, Magrane M, Ahmad S, Alpi E, et al. UniProt: the Universal Protein Knowledgebase in 2023. Nucleic Acids Res. 2023 Jan 6;51(D1):D523–31.

38. Goutelle S, Maurin M, Rougier F, Barbaut X, Bourguignon L, Ducher M, et al. The Hill equation: a review of its capabilities in pharmacological modelling. Fundam Clin Pharmacol. 2008 Dec 28;22(6):633–48.

39. PROCEEDINGS OF THE PHYSIOLOGICAL SOCIETY: January 22, 1910. J Physiol. 1910 Dec;40(suppl).

40. Sheu SY, Yang DY, Selzle HL, Schlag EW. Energetics of hydrogen bonds in peptides. Proceedings of the National Academy of Sciences. 2003 Oct 28;100(22):12683–7.

41. Nakagawa H, Tamada T. Hydration and its Hydrogen Bonding State on a Protein Surface in the Crystalline State as Revealed by Molecular Dynamics Simulation. Front Chem. 2021 Oct 18;9.

42. Jenkins CL, Vasbinder MM, Miller SJ, Raines RT. Peptide Bond Isosteres:  Ester or ( *E* )-Alkene in the Backbone of the Collagen Triple Helix. Org Lett. 2005 Jun 1;7(13):2619–22.

43. Boryskina OP, Bolbukh TV, Semenov MA, Gasan AI, Maleev VYa. Energies of peptide–peptide and peptide–water hydrogen bonds in collagen: Evidences from infrared spectroscopy, quartz piezogravimetry and differential scanning calorimetry. J Mol Struct. 2007 Feb;827(1–3):1–10.

44. Zhang X, Xu S, Shen L, Li G. Factors affecting thermal stability of collagen from the aspects of extraction, processing and modification. Journal of Leather Science and Engineering. 2020 Aug 5;2(1):19.

45. Jiang L, Kuhlman B, Kortemme T, Baker D. A “solvated rotamer” approach to modeling water‐mediated hydrogen bonds at protein–protein interfaces. Proteins: Structure, Function, and Bioinformatics. 2005 Mar 13;58(4):893–904.

46. Cutini M, Bocus M, Ugliengo P. Decoding Collagen Triple Helix Stability by Means of Hybrid DFT Simulations. J Phys Chem B. 2019 Aug 29;123(34):7354–64.

47. Biswal S, Agmon N. Collagen Structured Hydration. Biomolecules. 2023 Dec 4;13(12):1744.

48. Ahmed MH, Spyrakis F, Cozzini P, Tripathi PK, Mozzarelli A, Scarsdale JN, et al. Bound Water at Protein-Protein Interfaces: Partners, Roles and Hydrophobic Bubbles as a Conserved Motif. PLoS One. 2011 Sep 22;6(9):e24712.

49. Rodier F, Bahadur RP, Chakrabarti P, Janin J. Hydration of protein–protein interfaces. Proteins: Structure, Function, and Bioinformatics. 2005 Jul 26;60(1):36–45.

50. Grant CA, Brockwell DJ, Radford SE, Thomson NH. Tuning the Elastic Modulus of Hydrated Collagen Fibrils. Biophys J. 2009 Dec;97(11):2985–92.

51. Tourell MC, Momot KI. Molecular Dynamics of a Hydrated Collagen Peptide: Insights into Rotational Motion and Residence Times of Single-Water Bridges in Collagen. J Phys Chem B. 2016 Dec 15;120(49):12432–43.

52. Ravikumar KM, Hwang W. Region‐specific role of water in collagen unwinding and assembly. Proteins: Structure, Function, and Bioinformatics. 2008 Sep 2;72(4):1320–32.

53. Bella J, Eaton M, Brodsky B, Berman HM. Crystal and Molecular Structure of a Collagen-Like Peptide at 1.9 Å Resolution. Science (1979). 1994 Oct 7;266(5182):75–81.

54. Nakasako M. Water–protein interactions from high–resolution protein crystallography. Philos Trans R Soc Lond B Biol Sci. 2004 Aug 29;359(1448):1191–206.

55. Janin J. Wet and dry interfaces: the role of solvent in protein–protein and protein–DNA recognition. Structure. 1999 Jan;7(12):R277–9.

56. Fersht AR, Shi JP, Knill-Jones J, Lowe DM, Wilkinson AJ, Blow DM, et al. Hydrogen bonding and biological specificity analysed by protein engineering. Nature. 1985 Mar;314(6008):235–8.

57. Park W, Lee KM, Lee BS, Kim YJ, Joo SH, Kwak SK, et al. Hydrogen‐Bond Free Energy of Local Biological Water. Angewandte Chemie International Edition. 2020 Apr 27;59(18):7089–96.

58. Wells HC, Sizeland KH, Kelly SJR, Kirby N, Hawley A, Mudie S, et al. Collagen Fibril Intermolecular Spacing Changes with 2-Propanol: A Mechanism for Tissue Stiffness. ACS Biomater Sci Eng. 2017 Oct 9;3(10):2524–32.

59. Xu D, Tsai CJ, Nussinov R. Hydrogen bonds and salt bridges across protein-protein interfaces. Protein Engineering Design and Selection. 1997 Sep 1;10(9):999–1012.

60. Jeschke G. DEER Distance Measurements on Proteins. Annu Rev Phys Chem. 2012 May 5;63(1):419–46.

61. Levy Y, Onuchic JN. WATER MEDIATION IN PROTEIN FOLDING AND MOLECULAR RECOGNITION. Annu Rev Biophys Biomol Struct. 2006 Jun;35(1):389–415.

62. Best RB, Zhu X, Shim J, Lopes PEM, Mittal J, Feig M, et al. Optimization of the Additive CHARMM All-Atom Protein Force Field Targeting Improved Sampling of the Backbone ϕ, ψ and Side-Chain χ _1_ and χ _2_ Dihedral Angles. J Chem Theory Comput. 2012 Sep 11;8(9):3257–73.

63. Lorentz HA. Ueber die Anwendung des Satzes vom Virial in der kinetischen Theorie der Gase. Ann Phys. 1881 Jan 16;248(1):127–36.

64. Jorgensen WL, Chandrasekhar J, Madura JD, Impey RW, Klein ML. Comparison of simple potential functions for simulating liquid water. J Chem Phys. 1983 Jul 15;79(2):926–35.

65. Pace CN, Fu H, Fryar KL, Landua J, Trevino SR, Shirley BA, et al. Contribution of Hydrophobic Interactions to Protein Stability. J Mol Biol. 2011 May;408(3):514–28.

66. CHOTHIA C. Hydrophobic bonding and accessible surface area in proteins. Nature. 1974 Mar 1;248(5446):338–9.

67. Wimley WC, White SH. Experimentally determined hydrophobicity scale for proteins at membrane interfaces. Nat Struct Mol Biol. 1996 Oct 1;3(10):842–8.

68. Honig B, Nicholls A. Classical Electrostatics in Biology and Chemistry. Science (1979). 1995 May 26;268(5214):1144–9.

69. Freudenberg U, Behrens SH, Welzel PB, Müller M, Grimmer M, Salchert K, et al. Electrostatic Interactions Modulate the Conformation of Collagen I. Biophys J. 2007 Mar;92(6):2108–19.

70. Kumar S, Nussinov R. Close-Range Electrostatic Interactions in Proteins. ChemBioChem. 2002 Jul 3;3(7):604.

71. Donald JE, Kulp DW, DeGrado WF. Salt bridges: Geometrically specific, designable interactions. Proteins: Structure, Function, and Bioinformatics. 2011 Mar 5;79(3):898–915.

72. Price WD, Jockusch RA, Williams ER. Is Arginine a Zwitterion in the Gas Phase? J Am Chem Soc. 1997 Dec 1;119(49):11988–9.

73. Scherbakov KA, Kondratiev MS, Samchenko AA, Kabanov A V., Komarov VM. The electronic structure properties of 20 L-amino acids in neutral and zwitterion forms: Quantum-chemical calculations. Biophysics (Oxf). 2016 May 16;61(3):361–72.

74. Wada A. The alpha-helix as an electric macro-dipole. Adv Biophys. 1976;1–63.

75. Cornell WD, Cieplak P, Bayly CI, Gould IR, Merz KM, Ferguson DM, et al. A Second Generation Force Field for the Simulation of Proteins, Nucleic Acids, and Organic Molecules. J Am Chem Soc. 1995 May 1;117(19):5179–97.

76. MacKerell AD, Bashford D, Bellott M, Dunbrack RL, Evanseck JD, Field MJ, et al. All-Atom Empirical Potential for Molecular Modeling and Dynamics Studies of Proteins. J Phys Chem B. 1998 Apr 1;102(18):3586–616.

77. Reif MM, Hünenberger PH, Oostenbrink C. New Interaction Parameters for Charged Amino Acid Side Chains in the GROMOS Force Field. J Chem Theory Comput. 2012 Oct 9;8(10):3705–23.

78. Laskowski RA, MacArthur MW, Moss DS, Thornton JM. PROCHECK: a program to check the stereochemical quality of protein structures. J Appl Crystallogr. 1993 Apr 1;26(2):283–91.

79. Nayek A, Sen Gupta PS, Banerjee S, Mondal B, Bandyopadhyay AK. Salt-Bridge Energetics in Halophilic Proteins. PLoS One. 2014 Apr 17;9(4):e93862.

80. Looyenga H. Dielectric constants of heterogeneous mixtures. Physica. 1965 Mar;31(3):401–6.

81. Asami K. Characterization of heterogeneous systems by dielectric spectroscopy. Prog Polym Sci. 2002 Oct;27(8):1617–59.

82. Markel VA. Introduction to the Maxwell Garnett approximation: tutorial. Journal of the Optical Society of America A. 2016 Jul 1;33(7):1244.

83. Kaatze U. The dielectric properties of water in its different states of interaction. J Solution Chem. 1997 Nov;26(11):1049–112.

84. Waks M. Proteins and peptides in water‐restricted environments. Proteins: Structure, Function, and Bioinformatics. 1986 Jan 3;1(1):4–15.

85. Chandler D. Interfaces and the driving force of hydrophobic assembly. Nature. 2005 Sep 28;437(7059):640–7.

86. Lum K, Chandler D, Weeks JD. Hydrophobicity at Small and Large Length Scales. J Phys Chem B. 1999 Jun 1;103(22):4570–7.

87. Southall NT, Dill KA, Haymet ADJ. A View of the Hydrophobic Effect. J Phys Chem B. 2002 Jan 1;106(3):521–33.

88. Maroudas A, Wachtel E, Grushko G, Katz EP, Weinberg P. The effect of osmotic and mechanical pressures on water partitioning in articular cartilage. Biochimica et Biophysica Acta (BBA) - General Subjects. 1991 Mar;1073(2):285–94.

89. Hu Q, Ecker M. Overview of MMP-13 as a Promising Target for the Treatment of Osteoarthritis. Int J Mol Sci. 2021 Feb 9;22(4):1742.

90. Depalle B, Qin Z, Shefelbine SJ, Buehler MJ. Influence of cross-link structure, density and mechanical properties in the mesoscale deformation mechanisms of collagen fibrils. J Mech Behav Biomed Mater. 2015 Dec;52:1–13.

91. Adouni M, Dhaher YY. A multi-scale elasto-plastic model of articular cartilage. J Biomech. 2016 Sep;49(13):2891–8.

92. Hashemi J, Chandrashekar N, Slauterbeck J. The mechanical properties of the human patellar tendon are correlated to its mass density and are independent of sex. Clinical Biomechanics. 2005 Jul;20(6):645–52.

93. Maganaris CN, Paul JP. *In vivo* human tendon mechanical properties. J Physiol. 1999 Nov 7;521(1):307–13.

94. Oinas J, Ronkainen AP, Rieppo L, Finnilä MAJ, Iivarinen JT, van Weeren PR, et al. Composition, structure and tensile biomechanical properties of equine articular cartilage during growth and maturation. Sci Rep. 2018 Jul 27;8(1):11357.

95. Williamson AK, Chen AC, Masuda K, Thonar EJ‐ MA, Sah RL. Tensile mechanical properties of bovine articular cartilage: Variations with growth and relationships to collagen network components. Journal of Orthopaedic Research. 2003 Sep;21(5):872–80.

96. Akizuki S, Mow VC, Müller F, Pita JC, Howell DS, Manicourt DH. Tensile properties of human knee joint cartilage: I. Influence of ionic conditions, weight bearing, and fibrillation on the tensile modulus. Journal of Orthopaedic Research. 1986 Jan 18;4(4):379–92.

97. Mäkelä JTA, Huttu MRJ, Korhonen RK. Structure–function relationships in osteoarthritic human hip joint articular cartilage. Osteoarthritis Cartilage. 2012 Nov;20(11):1268–77.

98. Eyre DR, Dickson IR, Van Ness K. Collagen cross-linking in human bone and articular cartilage. Age-related changes in the content of mature hydroxypyridinium residues. Biochemical Journal. 1988 Jun 1;252(2):495–500.
